# ﻿Revision of *Immersaria* and a new lecanorine genus in Lecideaceae (lichenised Ascomycota, Lecanoromycetes)

**DOI:** 10.3897/mycokeys.87.72614

**Published:** 2022-02-15

**Authors:** Cong-Miao Xie, Li-Song Wang, Zun-Tian Zhao, Yan-Yun Zhang, Xin-Yu Wang, Lu-Lu Zhang

**Affiliations:** 1 Key Laboratory of Plant Stress Research, College of Life Sciences, Shandong Normal University, Jinan, Shandong, 250014, China; 2 CAS Key Laboratory for Plant Diversity and Biogeography of East Asia, Kunming Institute of Botany, Heilongtan, Kunming, Yunnan, 650204, China; 3 Institute of Environment and Ecology, Shandong Normal University, Jinan, Shandong, 250014, China

**Keywords:** China, generic classification, lecanorine apothecia, lichen, phylogeny, taxonomy

## Abstract

The species *Immersariacupreoatra* has been included in *Bellemerea*. This caused us to reconsider the relationships between *Bellemerea* and the lecanorine species of *Immersaria* and to question the monophyly of *Immersaria*. Amongst 25 genera of the family Lecideaceae, most have lecideine apothecia, the exceptions being *Bellemerea* and *Koerberiella*, which have lecanorine apothecia. According to previous classifications, *Immersaria* included species with both lecanorine and lecideine apothecia. A five-loci phylogenetic tree (nrITS, nrLSU, RPB1, RPB2, and mtSSU) for Lecideaceae showed that *Immersaria* was split into two clades: firstly, all the lecideine apotheciate species and secondly, all the lecanorine apotheciate species. The latter clade was closely related to the remaining lecanorine apotheciate genera: *Bellemerea* and *Koerberiella*. Therefore, the genus concept of *Immersaria* is revised accordingly and a new lecanorine genus *Lecaimmeria* is proposed. Furthermore, four new species for *Immersaria* and seven new species and three new combinations for the new genus *Lecaimmeria* are proposed. Keys to *Immersaria* and the new genus *Lecaimmeria* are provided.

## ﻿Introduction

The lichen genus *Immersaria* Rambold & Pietschm. (Rambold 1989) was originally split from the genus *Lecidea* Ach. in order to accommodate the species *Immersariaathroocarpa* (Ach.) Rambold & Pietschm. The genus *Immersaria* was characterised by its brown thallus with an epinecral layer, a pruinose margin and an amyloid medulla, immersed apothecia with a somewhat reduced proper margin and *Porpidia*-type asci with eight, simple, halonate ascospores (Rambold 1989). Subsequently, Calatayud and Rambold (1998) enlarged the scope of the genus by including the lecanorine species, *Immersariamehadiana* Calat. & Rambold and *I.cupreoatra* (Nyl.) Calat. & Rambold, based on morphological characters only. Currently, eight species of *Immersaria* are known worldwide (Lücking et al. 2017), three of which have lecanorine apothecia. Four of these species were previously reported from China (Hertel 1977; Zhang et al. 2015).

The species *Immersariacupreoatra* (Nyl.) Calat. & Rambold (= *Lecanoracupreoatra* Nyl.) was previously included in *Bellemerea* Hafellner & Cl. Roux (Clauzade and Roux 1984), then into *Immersaria* by Calatayud and Rambold (1998). This caused us to reconsider the relationships between *Bellemerea* and the lecanorine species of *Immersaria* and to question the monophyly of *Immersaria*. The family Lecideaceae Chevall originally included all the crustose lecideoid genera, but now only 25 genera have been retained. Most of these are monospecific genera or small genera with under five species (Fryday and Hertel 2014; McCune et al. 2017). Most genera in Lecideaceae have lecideine apothecia. Three exceptions are *Bellemerea*, *Immersaria* and *Koerberiella* Stein, which have lecanorine apothecia. Only *Immersaria* has both lecanorine and lecideine apothecia, according to the previous circumscription (Calatayud and Rambold 1998; Valadbeigi et al. 2011). Calatayud and Rambold (1998) indicated that the presence of “two types of ascomata” represent different stages of ontogeny. However, there was no molecular evidence that could clarify the species-level phylogenetic relationships within *Immersaria*. In the two-loci phylogenetic tree of Buschbom and Mueller (2004), the lecideine species *Immersariausbekica* (Hertel) M. Barbero, Nav.-Ros. & Cl. Roux was related to *Lecideatessellata* Flörke. However, because only two loci of one lecideine species were included, this tree was insufficient to clarify the relationship of the lecanorine apotheciate species in *Immersaria*.

In this study, a phylogenetic tree of Lecideaceae, based on five loci, is established in order to verify the monophyly of *Immersaria*. The results show that *Immersaria* is split into two clades. One clade includes all the lecideine apotheciate species, which is sister to *Lecideatessellata*, *L.auriculata* Th. Fr., *Cyclohymeniaepilithica* McCune & M.J. Curtis and the *Porpidiaalbocaerulescens* group and the *Porpidiaspeirea* group. The second clade contains all the lecanorine apotheciate species and is closely related to the rest of the lecanorine apotheciate genera within this family: *Bellemerea* and *Koerberiella*. Therefore, the genus concept of *Immersaria* is revised, retaining only the species with lecideine apothecia. The lecanorine species of *Immersaria* are excluded and proposed as a new genus, *Lecaimmeria* C.M. Xie, Lu L. Zhang & Li S. Wang. Furthermore, four new species for *Immersaria* and seven new species and three new combinations for the new genus *Lecaimmeria* are proposed, based on the four-loci phylogenetic trees. Keys to *Immersaria* and the new genus are provided below.

## ﻿Methods

### ﻿Morphological analysis

All the materials for this study were collected in mainland China, mostly from the Qinghai-Tibetan Plateau, during the authors’ participation in The Second Tibetan Plateau Scientific Expedition and Research Program. These specimens were stored in the Herbarium of the Kunming Institute of Botany, Chinese Academy of Sciences (KUN) and the Lichen Section of the Botanical Herbarium, Shandong Normal University (SDNU). Type specimens were loaned from the University of Helsinki (H) and Universität Wien (WU). High-resolution photographs of type specimens were provided by the curators of H or obtained from the website Global Plants (https://plants.jstor.org/). Morphological descriptions were made from under a dissecting microscope COIX. Anatomical descriptions were based on observations made from hand-cut sections, mounted in water and using a NIKON microscope. Usually, twenty ascospores were measured and the values of measurement means smallest measured-largest measured, with outlying values in brackets. Photographs were captured with a NIKON Eclipse 50i microscope, equipped with a NIKON digital camera (DSFi2 high-definition colour camera head, NIKON, Japan). The specimens were tested with a 10% aqueous solution of potassium hydroxide (K), a solution of aqueous sodium hypochlorite (C) and 3% Lugol’s iodine (I) in the medulla and the surface of the thallus. Secondary metabolites of all the specimens were examined by thin-layer chromatography (TLC) methods, using Solvents A, B and C, as described by Orange et al. (2001).

### ﻿Phylogenetic analysis

Molecular analysis was carried out on the selected specimens. Genomic DNA was extracted from dry or fresh specimens using a DNAsecure Plant Kit (Tiangen), following the manufacturer’s instructions. Five gene loci were amplified by using the following primers: ITS1F (Larena et al. 1999), ITS4 (White et al. 1990), LR0R (Rehner and Samuels 1994), LR5 (Vilgalys and Hester 1990), gRPB1a (Stiller and Hall 1997), fRPB1c (Matheny et al. 2002), RPB2–6f, RPB2–7cr (Liu et al. 1999), mrSSU1 and mrSSU3R (Zoller et al. 1999). The 25 μl PCR mixture consisted of 2 μl DNA, 1 μl of each primer, 12.5 μl 2 × Taq PCR MasterMix (Aidlab) (Taq DNA Polymerase [0.1 unit/ml]; 4 mM MgCl_2_; and 0.4 mM dNTPs) and 8.5 μl ddH_2_O. Conditions for PCR of nrITS, nrLSU and mtSSU were set for an initial denaturation at 94 °C for 10 min, followed by 34 cycles of denaturation at 95 °C for 45 s, annealing at 50 °C for 45 s, extension at 72 °C for 90 s and a final extension at 72 °C for 10 min. For RPB1 and RPB2, the parameters were set to an initial denaturation at 94 °C for 10 min, followed by 34 cycles of denaturation at 95 °C for 45 s, annealing at 52 °C for 50 s, extension at 72 °C for 60 s and a final extension at 72 °C for 5 min. The PCR products were sequenced using Sanger technology by the company of Tsingke Biological Technology (Beijing).

The raw sequences were assembled and edited using SeqMan v.7.0 (DNAstar packages). Sequences extracted from new materials with each gene locus were aligned with additional sequences that were available from GenBank (Suppl. material 1: Table S1), by using MEGA v.10.0 and an online version of MAFFT v.7.0 to generate nrITS-nrLSU-RPB1-RPB2-mtSSU or nrITS-nrLSU-RPB1-RPB2 matrices. The five or four gene matrices were combined by SequenceMatrix v.1.7.8. and the concatenated alignments were estimated by PartitionFinder 2 (Lanfear et al. 2017), based on the Bayesian Information Criterion (BIC), to find the most appropriate nucleotide substitution model for each of the five loci.

Phylogenetic relationships were inferred using Bayesian Inference (BI) and Maximum Likelihood (ML). ML analyses were performed with RAxMLHPC using the general time reversible model of nucleotide substitution with the gamma model of rate heterogeneity (GTRGAMMA or GTRCAT). The analyses were run with a rapid bootstrap analysis using 1000 replicates with data partitioned. The Bayesian method was performed with MrBayes v.3.1.2 (Huelsenbeck and Ronquist 2001). Four Markov chains were run with 2 million generations for each dataset and trees were sampled every 100 generations. It was ensured that the average standard deviation of split frequencies was lower than 0.01. Posterior probabilities above 0.9 and bootstrap support above 70% were considered significant supporting values. All the trees were visualised with FigTree v. 1.4.0 (Rambaut 2012).

## ﻿Results

**Figure 1.**
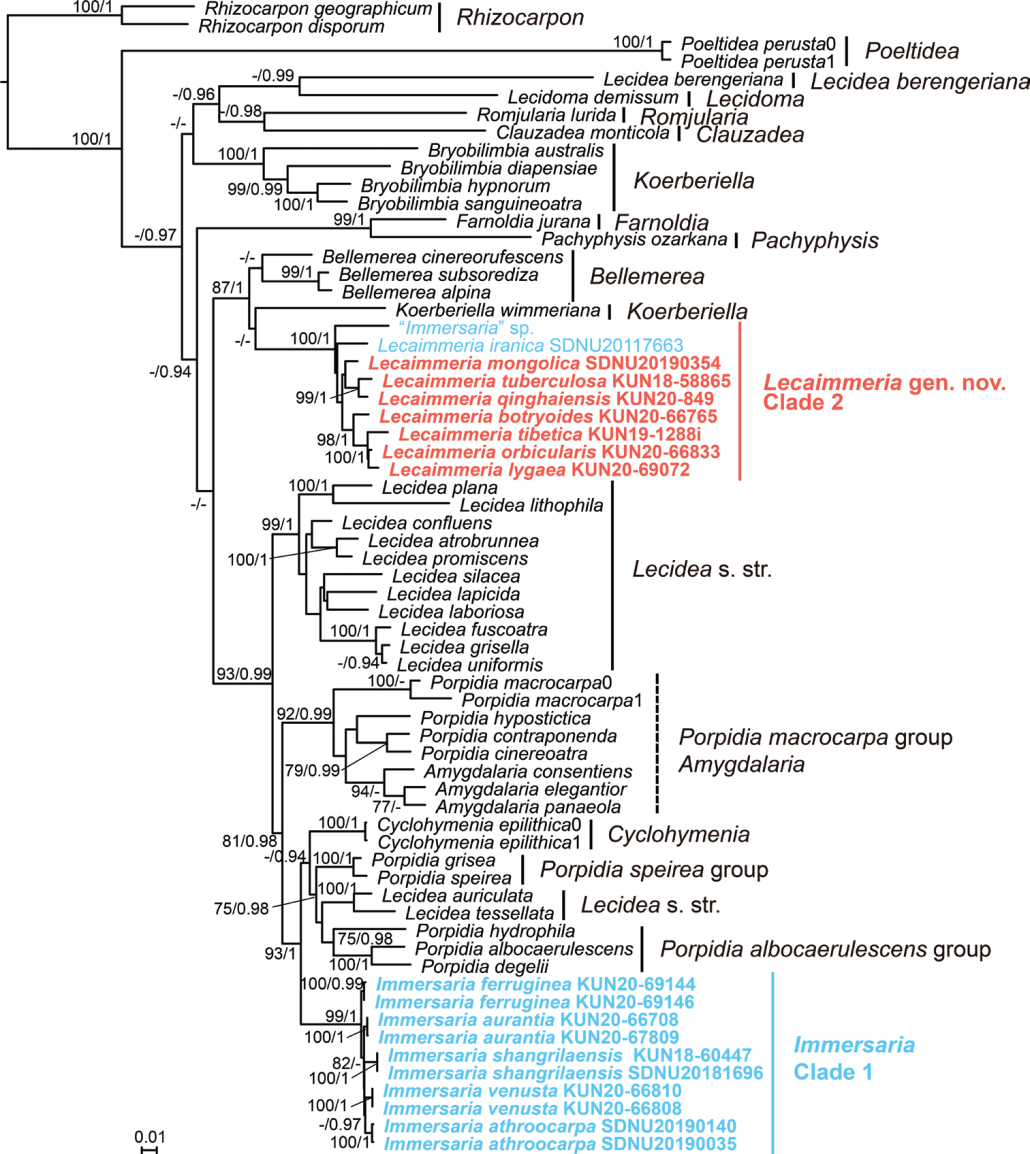
Phylogenetic tree constructed from Maximum Likelihood analyses in Lecideaceae, based on the concatenated nrITS-nrLSU-RPB1-RPB2-mtSSU dataset. Maximum Likelihood bootstrap probabilities above 70% (left) and Bayesian Inference posterior probabilities above 0.9 (right) are given at the nodes.

A total of 172 sequences of the nrITS, nrLSU, RPB1, RPB2, and mtSSU were generated from 61 specimens representing 57 species. Although the five-loci tree only poorly resolved the hierarchy of genera within the family Lecideaceae and the split between the lecanorine and lecideine genera of Lecideaceae was without robust support, nonetheless the results revealed that the genus *Immersaria* was not a monophyletic lineage. Rather, it was divided into two distant and well-supported lineages: clade 1 which contained the lecideine apotheciate species and clade 2 which contained the lecanorine apotheciate species (Fig. 1).

Clade 1, together with *Amygdalaria* Norman, *Cyclohymenia* McCune & M.J. Curtis, *Lecidea* s str. and *Porpidia* Körb. (Fig. 1) formed a well-supported clade (93%

MLBS and 0.99 PP), all of which have lecideine apothecia. *Amygdalaria*, *Porpidia* and *Lecidea* s str. were nested together, which was consistent with the results of previous research (Buschbom and Mueller 2004; Fryday et al. 2014). However, the relationships between these genera still need further research. There was a high level of support for a monophyletic lineage of lecideine apotheciate species of *Immersaria*, with these being sister to the *Lecideatessellata*, *L.auriculata*, *Cyclohymenia*, the *Porpidiaalbocaerulescens* group and the *Porpidiaspeirea* group. The type species of *Immersaria* (*I.athroocarpa*) was included in the lineage. Thus, only those *Immersaria* species with lecideine apothecia belong to *Immersaria* s. str. This revised concept of the genus *Immersaria* is as follows: a glossy surface of thallus with an epinecral layer, immersed lecideine apothecia with a reduced margin and *Porpidia*-type asci with halonate ascospores.

There was also a high level of support for clade 2 as a monophyletic lineage (100% MLBS and 1.00 PP), which was clustered with other genera of *Lecideales* with lecanorine apothecia: *Bellemerea* and *Koerberiella* (Fig. 1). *Bellemerea* could be distinguished from Clade 2 by its amyloid ascospores and *Koerberiella* by its adnate apothecia. Although the topology of clade 2 for *Bellemerea* and *Koerberiella* is not robust, there are conspicuous differences in their morphology and significant differences between the bases in their nucleotide sequences. Since clade 2 is monophyletic with strong support, a new genus, *Lecaimmeria*, is proposed to accommodate clade 2. The new genus has immersed lecanorine apothecia with a white margin and a distinct plectenchyma developed on top of the orange epihymenium.

**Figure 2.**
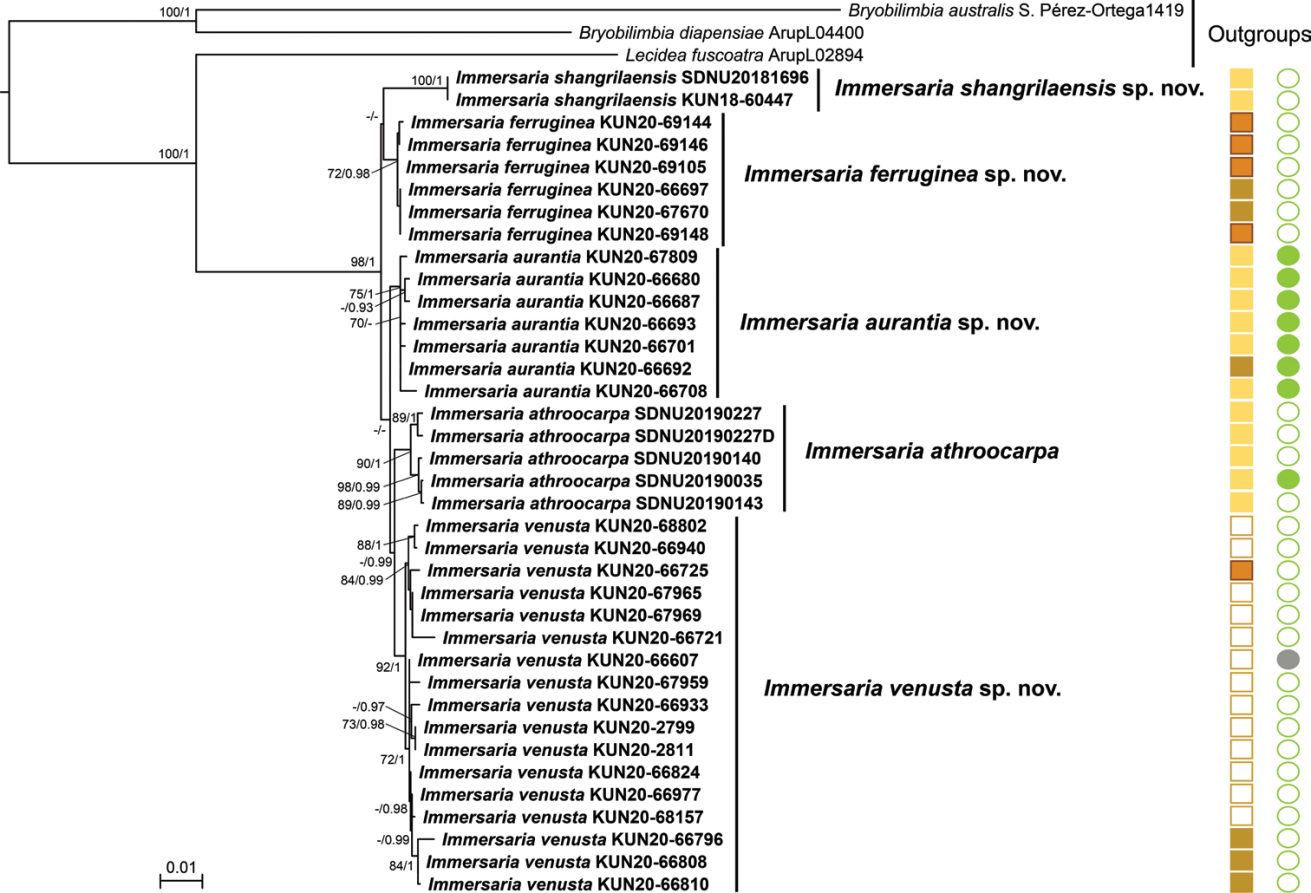
Phylogenetic tree constructed from Maximum Likelihood analyses in *Immersaria*, based on the concatenated nrITS-nrLSU-RPB1-RPB2 dataset. Maximum Likelihood bootstrap probabilities above 70% (left) and Bayesian Inference posterior probabilities above 0.9 (right) are given at the nodes. Solid brown rectangle: thallus brown; solid orange rectangle: thallus yellow brown to orange brown; solid red rectangle: thallus rusty; hollow brown rectangle: thallus pale yellow brown. Solid green circle: green epihymenium; solid grey circle: without apothecia; hollow green circular: brown epihymenium.

Two additional phylogenetic trees were constructed, based on four loci (nrITS, nrLSU, RPB1, and RPB2), in order to assess the phylogenetic position of species within *Immersaria* and *Lecaimmeria*, respectively. The phylogenetic tree of *Immersaria* was comprised of one highly supported clade with five separate lineages, based on 105 sequences from 37 specimens (Fig. 2). All the species with brown, orange, irregular or aggregate thalli formed respective monophyletic lineages. *Immersariashangrilaensis* C.M. Xie & Lu L. Zhang formed a well-supported clade and the aggregate areolae clearly distinguished *I.shangrilaensis* from other species. *Immersariaferruginea* C.M. Xie & Li S. Wang also formed a well-supported clade and differed from other species by its greyish-brown thallus. It seems that *Immersariashangrilaensis* is sister to *I.ferruginea*, but the nodes were without support. In addition, the morphology is distinct between *Immersariashangrilaensis* and *I.ferruginea*. The robust lineage *Immersariaaurantia* C.M. Xie & Li S. Wang was distinguished by its irregular, conspicuously orange thallus and green epihymenium. *Immersariaathroocarpa* was sister to *I.venusta* C.M. Xie & Xin Y. Wang, but differed in its convex, polygon areolae and densely crowded apothecia.

**Figure 3.**
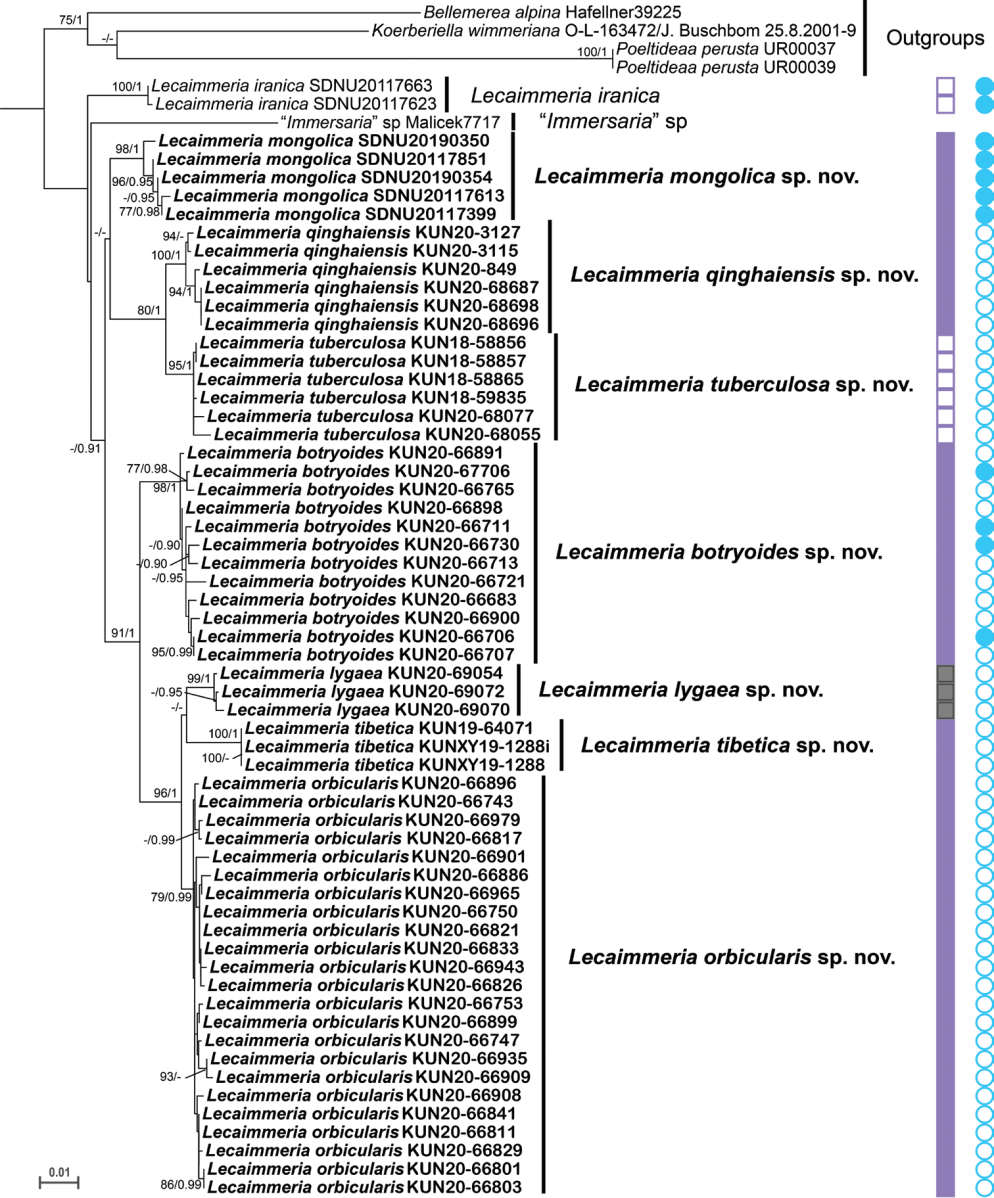
Phylogenetic tree constructed from Maximum Likelihood analyses in *Lecaimmeria*, based on the concatenated nrITS-nrLSU-RPB1-RPB2 dataset. Maximum Likelihood bootstrap probabilities above 70% (left) and Bayesian Inference posterior probabilities above 0.9 (right) are given at the nodes. Solid purple rectangle: areolae margin white; solid grey rectangle: areolae margin black; hollow purple rectangle: areolae margin jade green pigmented. Solid blue circle gyrophoric acid; hollow blue circle: none.

The phylogenetic tree of *Lecaimmeria* was comprised of one well-supported clade with nine separate lineages, based on 140 sequences from 61 specimens (Fig. 3). “*Immersaria*” sp. and *Lecaimmeriairanica* (Valadb., Sipman & Rambold) C.M. Xie comprised the basal group. “*Immersaria*” sp. has only been recorded from Macedonia and *Lecaimmeriairanica* has been recorded from Inner Mongolia in China and from Iran. *Lecaimmeriatuberculosa* C.M. Xie & Xin Y. Wang was sister to *L.qinghaiensis* C.M. Xie & Li S. Wang, but conspicuously differed in its tuberculiform conidiomata. *Lecaimmeriamongolica* C.M. Xie & Lu L. Zhang formed a well-supported monophyletic lineage and its population was mainly recognised by its orange, irregular areolae and gyrophoric acid content. *Lecaimmeriabotryoides* C.M. Xie & Li S. Wang formed a highly supported sister group to *L.orbicularis* C.M. Xie & Lu L. Zhang, *L.lygaea* C.M. Xie & Lu L. Zhang and *L.tibetica* C.M. Xie & Xin Y. Wang, but differed in its crowded apothecia. *Lecaimmeriaorbicularis* formed a highly supported sister group to *L.lygaea* and *L.tibetica*, but differed in its round apothecia and the white margin of the apothecia. *Lecaimmerialygaea* was seemingly sister to *L.tibetica* and differed in its areolae having a black margin and with a well-developed prothallus between areolae.

## ﻿Discussion

### ﻿Revised boundaries of *Immersaria*

Formerly, the boundaries for *Immersaria* species were: lecanorine or lecideine type of immersed apothecia, production of confluentic acid and gyrophoric acid and ostiole or stellate shapes of conidiomata. However, these characters were not good characters by which to distinguish this genus. The lecanorine species *Immersariacupreoatra* was previously included in *Bellemerea*. Based on many specimens from China, it was also discovered that the ostiole or stellate shapes of conidiomata appeared in different stages of ontogeny. The main substances produced in the genus are confluentic acid and gyrophoric acid; confluentic acid only occurs in lecideine species, whereas gyrophoric acid appears in lecanorine species, with the exception of one lecideine species *Immersariausbekica.* Furthermore, these characters, the types of apothecia and the shapes of conidiomata could not be applied as proper delimitations to classify species within *Immersaria*, neither were they supported by the phylogeny.

The five-loci based analysis (Fig. 1) was incompatible with previous circumscriptions of the genus *Immersaria*, the members of which in this study are defined by their lecideine immersed apothecia, brown areolae with an epinecral layer and brown/green epihymenium without a plectenchyma. Consequently, a new genus, *Lecaimmeria*, is established to accommodate the excluded lecanorine species. The new taxonomic system, proposed here, revised the classification boundaries between *Immersaria* and *Lecaimmeria*, but it may still be difficult to distinguish between them in cases when apothecia are absent. In this case, they could be distinguished by the substances produced or by molecular methods.

### ﻿Diagnostic traits within species of *Immersaria* and *Lecaimmeria*

Species of *Immersaria* could be identified by their different thallus colours (indicated in Fig. 2). *Immersariaferruginea* has a conspicuously greyish-brown thallus, whereas *I.athroocarpa*, *I.aurantia*, *I.shangrilaensis* and *I.venusta* have a reddish-brown thallus. *Immersariaathroocarpa* (indicated in Fig. 2) is the species that mostly has a green epihymenium, whereas the other species mostly have a brown epihymenium. Almost all these species contain confluentic acid, which is often accompanied by 2’-O-methylmicrophyllinic acid. Planaic acid, which is newly reported from this genus, is only presented in specimens of *Immersariaaurantia*, *I.shangrilaensis* and *I.venusta*. All the characters discussed above were supported by the phylogeny, thus could be used as key characters to differentiate species in *Immersaria*.

Species of the new genus *Lecaimmeria* could be delimited by the colours of their areolae and margins, the existence of an apothecial margin and usually by the lack of substances. The margin of areolae (indicated in Fig. 3) was usually white, but rarely black or jade green. *Lecaimmerialygaea* could be easily distinguished by the black margin of the areolae. The jade green margin occurs in *Lecaimmeriatuberculosa*, which grows on Qilian jade. The areolae margin of *Lecaimmeriaqinghaiensis* is white, but is occasionally pigmented with very slightly green colour. The margin of the apothecia is absent in *Lecaimmeriatuberculosa* and *L.iranica*, whereas the apothecia of the other species have white margins. Most species of *Lecaimmeria* lack secondary metabolites, while gyrophoric acid was detected only in *Lecaimmeriabotryoides*, *L.iranica* and *L.mongolica* (indicated in Fig. 3). In addition, an orange thallus appeared only in *Lecaimmeriamongolica* and *L.tibetica*, whereas the remaining species were brownish.

## ﻿Taxonomy

### ﻿ Immersaria

Taxon classificationFungiLecidealesLecideaceae

Rambold & Pietschm., Bibliotheca Lichenologica 34: 239 (1989).

A91C4667-20CA-5935-A8E3-BDC76DEDE0E5

#### Type species.

*Immersariaathroocarpa* (Ach.) Rambold & Pietschm., in Rambold, Biblioth. Lichenol. 34: 240 (1989).

#### Description.

Thallus crustose, yellow-brown, red-brown, orange-brown or brown, sometimes rust coloured, continuous; areolae irregular or tending to rectangular, with a glossy surface (*atrobrunnea*-type) caused by a layer of dead, colourless cells above the upper cortex, areolae sometimes aggregate with black prothallus and forming larger areolae; margin pruinose; prothallus distinct at the margin of thallus or absent. Upper cortex orange pigmented; epinecral layer colourless; algal layer continuous; medulla filled with grey granules. Apothecia lecideine, immersed, sometimes aggregate, round or irregular; disc black, flat, less concave, sometimes slightly raised, often poorly developed in section, pruinose or not; margin reduced. Exciple almost absent, sometimes developed, brown. Hymenium colourless; paraphyses simple, rarely branched, anastomosing or not; epihymenium brown, green or brown green, without plectenchyma; subhymenium colourless, sometimes pale brown; hypothecium pale brown to brown. Asci *Porpidia*-type, cylindrical, eight-spored; ascospores ellipsoid, halonate, non-amyloid. Conidiomata present or not, immersed, linear or stellate, black, margin pruinose; conidia bacilliform.

#### Chemistry.

Thallus K–, C–. Medulla I+ violet. Confluentic acid, often accompanied with 2’-O-methylmicophyllinic acid, planaic acid or no substances detected by TLC. The compound planaic acid is newly reported in this genus.

#### Ecology and distribution.

In China, growing on bare rock, sandstone or granite, from elevations of 3800 to 4500 m in the alpine zone of west China and elevations of 1200 to 1900 m in the steppe of north China. Worldwide distribution.

#### Notes.

Species with lecanorine apothecia were previously included in *Immersaria* (Calatayud and Rambold 1998; Valadbeigi et al. 2011), but the five-loci phylogenetic analysis excluded these species from *Immersaria.* This exclusion entails a restricted concept of the genus. *Immersaria* is now defined by its orange-brown, yellow-brown, sometimes rusty coloured thallus, the amyloid medulla, the glossy surface of areolae with a pruinose margin, the black immersed lecideine apothecia with a reduced proper margin, the brown epihymenium and the *Porpidia*-type asci with eight halonate and non-amyloid ascospores. The members of this genus occur in alpine habitats.

Species of *Sporastatia* A. Massal. might be misidentified as members of *Immersaria* because of field observations of their glossy areolae and the immersed lecideine apothecia. However, they are characterised by multi-spored asci and their yellow-brown thallus. Additionally, *Miriquidica* Hertel & Rambold resembles *Immersaria* by its glossy areolae and the lecideine apothecia, but differs in its black brown thallus, its *Lecanora*-type asci with non-halonate ascospores and often containing miriquidic acid. The immersed apothecia of *Immersaria* may resemble *Aspicilia* A. Massal. and *Acarospora* A. Massal., but *Aspicilia* has a white or grey thallus, the *Aspicilia*-type asci with non-halonate ascospores; *Acarospora* has multi-spored asci.

Although four known species, *Immersariacarbonoidea* (J.W. Thomson) Esnault & Cl. Roux, *I.fuliginosa* Fryday, *I.olivacea* Calat. & Rambold and *I.usbekica*, currently lack molecular data, they are temporarily left in *Immersaria* due to their morphology which corresponds to that of *Immersaria.* Our morphological comparisons were based on high-resolution photographs of type materials and the original descriptions.

### ﻿ Immersaria athroocarpa

Taxon classificationFungiLecidealesLecideaceae

(Ach.) Rambold & Pietschm., in Rambold, Biblioth. Lichenol. 34: 240 (1989).

06C131E8-6ACC-53BB-82B6-E0688F77E39F

Figure 4a–e

#### Type.

Sweden [no locality, no date, no collector], H9508237 (H-Ach-lectotype!–designated in Hertel 1977). High-resolution photographs seen.

#### Description.

Thallus areolate, yellow-brown, orange-brown, continuous; areolae 0.2–1.0 mm across, often convex, regular polygons, tends to be squamalose at the margin, epruinose; margin pruinose; prothallus black, not distinct, sometimes absent. Upper cortex ca. 32.0 μm thick, yellow-brown; epinecral layer ca. 7.0 μm thick; algal layer ca. 82.0 μm thick, cells 8.0–10.0 × 7.5 μm in diam., ellipsoid. Apothecia frequent, densely crowded, immersed, 0.3–1.3 mm in diam.; disc black, rare pruinose, flat, epruinose; margin reduced. Exciple sometimes developed, 25.0–30.0 μm wide, brown. Hymenium 100–115 μm thick, colourless; paraphyses 1.0–2.0 μm wide, branched, not anastomosing; epihymenium 20.0–25.0 μm thick, brown, rarely green; subhymenium ca. 90.0 μm thick, colourless; hypothecium pale brown to brown. Asci *Porpidia*-type, cylindrical, eight-spored; ascospores 17.5–20.0 × 10.0 μm, ellipsoid, halonate. Conidiomata immersed, stellate, black, margin pruinose; conidia 7.5–10.0 × 1.0 μm in diam., bacilliform.

#### Chemistry.

Thallus K–, C–. Medulla I+ violet. Chemotype Ⅰ: Confluentic acid. Chemotype Ⅱ: Unknown substance.

#### Ecology and distribution.

In China, growing on granite in arid and semi-arid steppe habitats at elevations of 1200–1950 m. Worldwide distribution. This species is known from Inner Mongolia and Mt. Changbai (Hertel and Zhao 1982) in China.

#### Notes.

The lectotype grows on siliceous rock and contains several intact apothecia. The materials from Inner Mongolia are identical with the lectotype, based on comparisons with high-resolution photographs and the description given by Hertel (1977). It is, therefore, treated as *Immersariaathroocarpa* at present. Some Inner Mongolian materials contain an unknown substance, but form a well-supported clade with other materials. *Immersariaathroocarpa* is characterised by the convex, yellow-brown areolae and the large sizes of ascospores. In this genus, only this species has ascospores up to 20.0 μm long.

**Figure 4.**
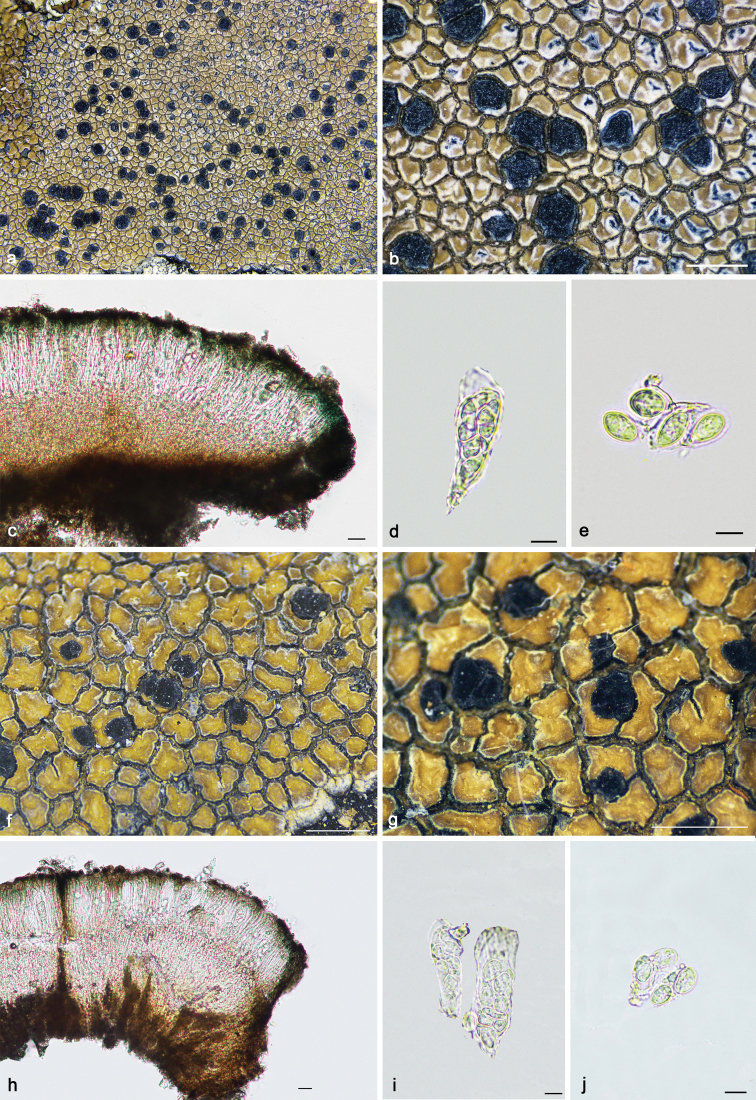
*Immersariaathroocarpa* (**a–e** SDNU20190227): **a–b** thallus **c** apothecial anatomy **d** ascus **e** ascospores. *I.aurantia* (**f–j**KUN XY19–1290): **f–g** thallus **h** apothecial anatomy **i** ascus **j** ascospores. Scale bars: 1 mm (**a–b, f–g**); 20 μm (**c, h**); 10 μm (**d–e, i–j**).

*Immersariausbekica* is similar to *I.athroocarpa* in its brown thallus and dense apothecia, but differs in its flat areolae, the brown epihymenium and the presence of confluentic acid and gyrophoric acid. By comparison with high-resolution photographs and the original descriptions (Hertel 1977) of *Immersariausbekica*, we discovered that previous reports of this species from China (Zhang et al. 2015) were due to misidentification. It is known from Algeria, Iran, Spain, and the USSR (Barbero et al. 1990).

#### Specimens examined (SDNU).

China. Inner Mongolia: Chifeng City, Balin Youqi, Hongshilazi, 1403.2 m elev., 44°13'N, 118°44'E, on rock, 2019, Ling Hu et al. SDNU20190035; Rongshen, Wangfengou, 1217.4 m elev., 44°16'N, 118°22'E, Ling Hu et al. SDNU20190140, SDNU20190143; Erlinba, 1915.2 m elev., 44°26'N, 118°41'E, Ling Hu et al. SDNU20190227.

### ﻿ Immersaria aurantia

Taxon classificationFungiLecidealesLecideaceae

C.M. Xie & Li S. Wang sp. nov.

F7AAD70F-29DE-5969-A457-3442492653BC

839738

Figure 4f–j

#### Etymology.

The name “aurantia” refers to the orange thallus.

#### Type.

China. Tibet: Sajia Co., Mula Village, 4752 m elev., 28°40'N, 88°45'E, on rock, 28 Jun 2019, Xin-Yu Wang et al. XY19–1814 (KUN-holotype).

#### Description.

Thallus areolate, orange, dark orange, pale orange to pale red-brown, continuous; areolae 0.7–1.3 mm across, flat, epruinose, irregular; margin thin pruinose; prothallus not seen. Upper cortex 25.0–45.0 μm thick, orange; epinecral layer (12.0–) 37.0–63.0 μm thick, uneven; algal layer 50.0–93.0 μm thick, cells 5.0–15.0 × 5.0–10.0 μm in diam., round to ellipsoid. Apothecia frequent, scattered, immersed or isolated from areolae, 0.3–1.3 mm in diam.; disc black, flat or concave, sometimes pruinose; margin reduced. Exciple sometimes developed, ca. 30.0 μm wide, brown. Hymenium 55.0–83.0 μm thick, colourless; paraphyses 2.0–3.0 μm wide, only branched and anastomosing at apex; epihymenium ca. 20.0 µm thick, green or green-brown; subhymenium colourless, not distinct or absent; hypothecium brown. Asci *Porpidia*-type, cylindrical, eight-spored; ascospores 8.0–15.0 × 5.0–7.5 μm in diam., ellipsoid, halonate. Conidiomata rare, immersed, oblate, black, margin white; conidia 7.5 × 1.0 μm, bacilliform.

#### Chemistry.

Thallus K–, C–. Medulla I + violet. Chemotype Ⅰ: Confluentic acid, often accompanied with 2’-O-methylmicrophyllinic acid. Chemotype Ⅱ: Planaic acid. Chemotype Ⅲ: none (rare).

#### Ecology and distribution.

In China, growing on rock at elevations of 3900–4300 m in the alpine zone. This species is known from Qinghai, Sichuan Province and Tibet of China.

#### Notes.

*Immersariaaurantia* is characterised by its distinct orange, irregular areolae and the mostly green epihymenium. *Immersariaathroocarpa* and *I.venusta* are similar to *I.aurantia*, but *I.athroocarpa* differs in the convex, regularly polygonal areolae and the more crowded apothecia; *I.venusta* differs in having yellow-brown, often rusty, cracked areolae and flat apothecia. Additionally, confluentic acid and planiaic acid do not appear simultaneously in *Immersariaaurantia*, whereas *I.venusta* always contains both compounds.

#### Specimens examined (KUN).

China. Qinghai Province: Banma Co., 3933 m elev., 32°40'N, 100°48'E, on rock, 2020, Li-Song Wang et al. 20–66886, 3932 m elev., Li-Song Wang et al. 20–66897; Jiuzhi Co., Baiyu Village, 4285 m elev., 33°14'N, 100°58'E, Li-Song Wang et al. 20–67809. Sichuan Province: Rangtang Co., Mt. Haizi, 4223 m elev., 32°20'N, 101°25'E, on rock, 2020, Li-Song Wang et al. 20–66701, 4229 m elev., Li-Song Wang et al. 20–66693, 4217 m elev., Li-Song Wang et al. 20–66680, 4221 m elev., Li-Song Wang et al. 20–66692. Tibet: Changdu City, Mangkang Co., Luoni Village, 4145 m elev., 29°56'N, 98°33'E, on rock, 2020, Li-Song Wang et al. 20–69091, 4138 m elev., Li-Song Wang et al. 20–69091, 20–69094; Gatuo Town, 29°39'N, 98°35'E, 3831 m elev., Li-Song Wang et al. 20–69114, 3850 m elev., Li-Song Wang et al. 20–69122; Gongga Co., Jiangtang Town, 29°12'N, 90°38'E, 2019.7.23, 4560 m elev., Xin-Yu Wang et al. XY19–1287, 4556 m elev., XY19–1290; Sajia Co., Mula Village, 28°40'N, 88°45'E, 2019.7.28, 4752 m elev., Xin-Yu Wang et al. XY19–1814; Angren Co., Kerangla, 29°19'N, 87°01'E, 4530 m elev., Li-Song Wang et al. 19–63635.

### ﻿ Immersaria ferruginea

Taxon classificationFungiLecidealesLecideaceae

C.M. Xie & Li S. Wang sp. nov.

DAE670AA-5077-5132-B3B1-820539E5A95A

839739

Figure 5a–c

#### Etymology.

The name “ferruginea” refers to the rusty brown colour of the thallus.

#### Type.

China. Tibet: Changdu City, Mangkang Co., Quzika Village, 4093 m elev., 29°15'N, 98°40'E, on rock, 25 Sept 2020, Li-Song Wang et al. 20–69144 (KUN-holotype).

#### Description.

Thallus areolate, greyish-brown, continuous; areolae 0.5–1.3 mm across, flat, less often convex, rectangular to polygonal, epruinose; margin pruinose; prothallus black, not distinct. Upper cortex 50.0–68.0 μm thick, brown; epinecral layer 17.0–40.0 μm thick; algal layer 75.0–78.0 μm thick, cells (4.0–) 7.0–13.0 μm diam., round. Apothecia frequent, densely crowded, immersed, 0.7–1.3 mm in diam.; disc black, flat, pruinose; margin pruinose, slightly raised. Exciple sometimes developed, 25.0–28.0 μm wide, brown. Hymenium 57.0–100.0 μm thick, colourless; paraphyses 1.0–3.0 μm wide, not branched, anastomosing; epihymenium 15.0–33.0 μm thick, brown; subhymenium 25.0–63.0 μm thick, colourless to pale brown, rusty or dark pink; hypothecium pale brown. Asci *Porpidia*-type, cylindrical; ascospores rare, 7.5–10.0 × 5.0 μm in diam., ellipsoid, halonate. Conidiomata not seen.

**Figure 5.**
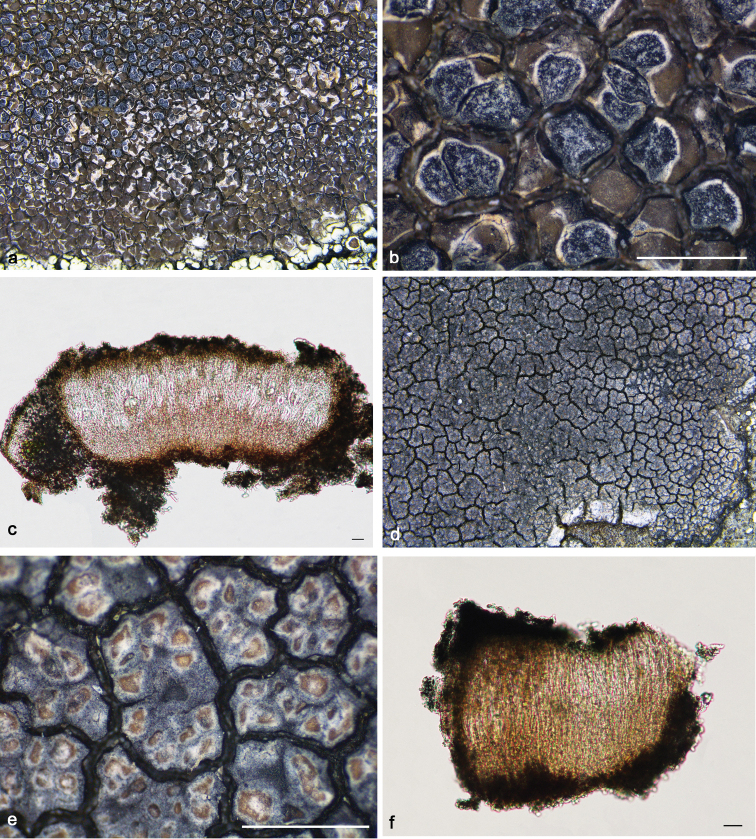
*Immersariaferruginea* (**a–c**KUN 20–69144): **a–b** thallus **c** apothecial anatomy. *I.shangrilaensis* (**d–f**KUN 18–60430): **d–e** thallus **f** apothecial anatomy. Scale bars: 1 mm (**a–b, d–e**); 20 μm (**c, f**).

#### Chemistry.

Thallus K–, C–. Medulla I+ violet. Confluentic acid, often accompanied with 2’-O-methylmicrophyllinic acid.

#### Ecology and distribution.

In China, growing on quartz sandstone or granite at elevations of 3800–4300 m in the alpine zone. This species is known from Sichuan Province and Tibet of China.

#### Notes.

*Immersariaferruginea* is characterised by its brown, rusty thallus, its densely crowded apothecia and its brown epihymenium. The morphology of *Immersariaferruginea* resembles *I.carbonoidea*, but the latter differs in its dark black-brown thallus containing norstictic acid and black-brown hypothecium.

#### Specimens examined (KUN).

China. Sichuan Province: Rangtang Co., Mt. Haizi, 4227 m elev., 32°20'N, 101°25'E, on rock, 2020, Li-Song Wang et al. 20–66697, 4221 m elev., Li-Song Wang et al. 20–67670. Tibet: Changdu City, Mangkang Co., Quzika Village, 4093 m elev., 29°15'N, 98°40'E, Li-Song Wang et al. 20–69144, 4101, Li-Song Wang et al. 20–69146, 4122 m elev., Li-Song Wang et al. 20–69148; Gatuo Town, 3848 m elev., 29°39'N, 98°35'E, Li-Song Wang et al. 20–69105.

### ﻿ Immersaria shangrilaensis

Taxon classificationFungiLecidealesLecideaceae

C.M. Xie & Lu L. Zhang sp. nov.

ABC02701-E5CD-568C-8D1B-B21930BA5BE8

839741

Figure 5d–f

#### Etymology.

The name “shangrilaensis” refers to the location at which the holotype was collected: “Shangri-La”, a county of Yunnan Province in China.

#### Type.

China. Yunnan Province: Shangri-La County., Mt. Hong Shan, 4363 m elev., 28°7'N, 99°54'E, on rock, 18 Aug 2018, Li-Song Wang et al. 18–60447 (KUN-holotype).

#### Description.

Thallus areolate, yellow-brown, orange-brown, often appears as greyish-brown, generally heavily pruinose, continuous, 5.7–10.0 cm across; areolae aggregated by 4–14 small areolae (often surrounded by black prothallus), small areolae up to 0.1 mm across, concave or flat, irregular, pruinose; margin pruinose; prothallus black, distinct. Upper cortex 32.0–50.0 μm thick, yellow-brown granules pigmented; epinecral layer 15.0–20.0 μm thick; algal layer 47.5–65.0 μm thick, cells 7.5–8.0 × 5.0 μm in diam., ellipsoid. Apothecia frequent, crowded, immersed or isolated from areolae, 0.3–0.8 mm in diam.; disc black, concave to flat, aggregated, cracked once mature, thin pruinose; margin reduced, slightly raised. Exciple almost absent. Hymenium 100.0–138.0 μm thick, colourless; paraphyses ca. 2.5 μm wide, branched, anastomosing or not; epihymenium ca. 15.0 μm thick, brown; subhymenium ca. 55.0 μm thick, colourless; hypothecium pale brown to brown. Asci *Porpidia*-type, cylindrical, eight-spored; ascospores 7.0–9.0 × 3.0–4.0 μm, ellipsoid, halonate (sometimes not distinct). Conidiomata immersed, oblate, black, margin heavily pruinose; conidia 7.5 × 1.0 μm, bacilliform.

#### Chemistry.

Thallus K–, C–. Medulla I+ violet. Confluentic acid, planaic acid and/or 2’-O-methylmicophyllinic acid.

#### Ecology and distribution.

In China, growing on granite at elevations of 4300–4500 m in the alpine zone. This species is known from Yunnan Province of China.

#### Notes.

The materials of *Immersariaathroocarpa* from the Shangri-La County of Yunnan Province are morphologically identical with the specimen Hertel (1977) reported from the same locality, but differ from the lectotype in its aggregate areolae, the aggregate apothecia and the smaller size of ascospores (7.0–9.0 × 3.0–4.0 μm). Based on the phenotypic and phylogenetic results, the material from Shangri-La is treated as a new species, *Immersariashangrilaensis*. It is characterised by its large thallus, up to 10.0 cm in diam., the aggregate areolae and apothecia and the small size of ascospores.

#### Specimens examined.

China. Yunnan Province: Shangri-La County, 4350–4500 m elev., on rock, 1915, Handel-Mazzetti no. 6945 = WU-Lichenes0037752 (WU); Mt. Hong Shan, 4363 m elev., 28°7'N, 99°54'E, on rock, 2018, Li-Song Wang et al. 18–60430 (KUN), Li-Song Wang et al. 18–60447 (KUN) 4503.1 m elev., Chun-Xiao Wang et al. SDNU20181696 (SDNU), 4361.9 m elev., Chun-Xiao Wang et al. SDNU20181675 (SDNU); Luquan Co., Mt. Jiaozixueshan, 3800 m elev., 2008, Hai-Ying Wang SDNU20082253 (SDNU); Lijiang City, Mt. Laojun, 3981 m elev., 26°37'N, 99°43'E, 2018, Li-Song Wang et al. 18–60555, 18–60602 (KUN).

### ﻿ Immersaria venusta

Taxon classificationFungiLecidealesLecideaceae

C.M. Xie & Xin Y. Wang sp. nov.

B17CABFE-2CFD-5099-B528-ADB037E575CB

839742

Figure 6a–d

#### Etymology.

The name “venusta” refers to the beautiful appearance of the thallus.

#### Type.

China. Qinghai Province: Maqing Co., Xueshan Village, 4187 m elev., 34°37'N, 99°42'E, on rock, 11 Sept 2020, Li-Song Wang et al. 20–67969 (KUN-holotype).

#### Description.

Thallus areolate, brown, orange-brown, more or less rusty, continuous; areolae 0.5–1.3 mm across, flat or slightly convex, irregular, tending to rectangular, often cracked, sometimes pruinose; margin pruinose; prothallus not seen. Upper cortex ca. 38.0 μm thick, yellow brown granules pigmented; epinecral layer ca. 12.0 μm thick; algal layer ca. 128.0 μm thick, cells 5.0–10.0 × 5.0–7.5 μm in diam., round to ellipsoid. Apothecia frequent, crowded, immersed or isolated from areolae, 0.6–1.0 mm in diam.; disc black, flat, epruinose; margin reduced, sometimes developed. Exciple sometimes developed, ca. 35.0 μm wide, brown. Hymenium 92.0–113.0 μm thick, colourless; paraphyses ca. 2.0 μm wide, anastomosing; epihymenium 27.5–30.0 μm thick, brown; subhymenium ca. 62.0 μm thick, colourless; hypothecium brown. Asci *Porpidia*-type, cylindrical, eight-spored; ascospores 10.0–12.5 × 5.0–7.5 μm, ellipsoid, halonate. Conidiomata immersed, linear, black, margin pruinose; conidia not seen.

**Figure 6.**
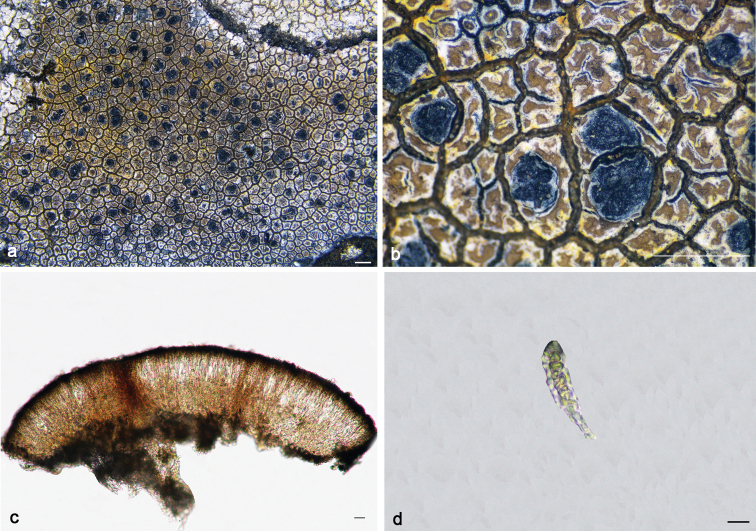
*Immersariavenusta* (**a–d**KUN 20–66725): **a–b** thallus **c** apothecial anatomy **d** ascus. Scale bars: 1 mm (**a–b**); 20 μm (**c**); 10 μm (**d**).

#### Chemistry.

Thallus K–, C–. Medulla I+ violet. Chemotype Ⅰ: Confluentic acid, often accompanied with 2’-O-methylmicrophyllinic acid. Chemotype Ⅱ: Planaic acid. Chemotype Ⅲ: none (rare).

#### Ecology and distribution.

In China, growing on quartz sandstone or granite at elevations of 3900–4300 m in the alpine zone. This species is known from Qinghai Province of China.

#### Notes.

*Immersariavenusta* is characterised by its yellow-brown, cracked areolae, its flat apothecia and brown epihymenium. It resembles *Immersariashangrilaensis* by its cracked areolae, but its areolae have the tendency to split into several patches, but not aggregate like those of *I.shangrilaensis. Immersariaathroocarpa* is similar to *I.venusta* in the brown appearance of its thallus and in forming a sister group in the phylogenetic tree, but it differs in its yellow brown thallus, convex areolae, densely crowded apothecia and larger ascospores (17.5–20.0 × 10.0 μm). *Immersariavenusta* is also similar to *I.aurantia* (see notes for *I.aurantia*). The brown thallus of *Immersariavenusta* possibly resembles that of *I.olivacea*, but the latter differs in its simple or one-septate ascospores, pyriform conidia and dark bluish-green epihymenium.

#### Specimens examined (KUN).

China. Qinghai Province: Maqing Co., Xueshan Village, 4187 m elev., 34°37'N, 99°42'E, on rock, 2020, Li-Song Wang et al. 20–67969, 20–67965; Banma Co., Yaertang Village, 3930 m elev., 32°42'N, 100°42'E, Li-Song Wang et al. 20–66940. Sichuan Province: Shiqu Co., Xinrong Village, 4043 m elev., 32°59'N, 98°19'E, on rock, 2020, Li-Song Wang et al. 20–68802; Rangtang Co., Mt. Haizi, 4246 m elev., 32°21'N, 101°24'E, Li-Song Wang et al. 20–66721, 20–66725.

#### Selected additional comparative material was examined.

*Bellemereaalpina* (Sommerf.) Clauzade & Cl. Roux Russia, Lps. Petsamo, Pummangin vuonon N-puoli, 1938, Räsänen, V., H9503269 (H); Lps. Petsamo, inter Vaitolahti et Kervanto, 1938, Räsänen, V., H9503270 (H).

*Bellemereacinereorufescens* (Ach.) Clauzade & Cl. Roux Finland, Ob. Simo. Anteroinen. Rantakivellä, 1920, Räsänen, V., H9503267 (H); Le. Enontekiö, Kirkonkylä, 1925, Kari, L.E., H9503268 (H).

#### High-resolution photographs seen.

*Immersariacarbonoidea* (J.W. Thomson) Esnault & Cl. Roux USA, Alaska, along the Pitmegea River, 15 miles upstream from Cape Sabine, 1958, Thomson, J.W., M0082171 (M-isotype!), G00126754 (G-isotype!).

*Immersariaolivacea* Calat. & Rambold Spain, Espana, Castelló: Benicàssim, Parreta Alta, 390 m elev., 1993, Calatayud, V., M0101779 (M-isotype!).

*Immersariausbekica* (Hertel) M. Barbero, Nav.-Ros. & Cl. Roux Algeria Algerie-Atlas Tellieu, Larba, Piste de Bougara á Tablat au S-E de l’arboretum de Meindja, 1985, Esnault, J., M0101787 (M-paratype!).

### ﻿ Lecaimmeria

Taxon classificationFungiLecidealesLecideaceae

C.M. Xie, Lu L. Zhang & Li S. Wang gen. nov.

F26E10D5-1229-5C2A-8D46-30EF3DAF1E3E

839743

#### Etymology.

The name “*Lecaimmeria*” refers to the immersed lecanorine apothecia of all known species.

#### Type species.

*Lecaimmeriaorbicularis* C.M. Xie & Lu L. Zhang, sp. nov.

#### Description.

**Figure 7.**
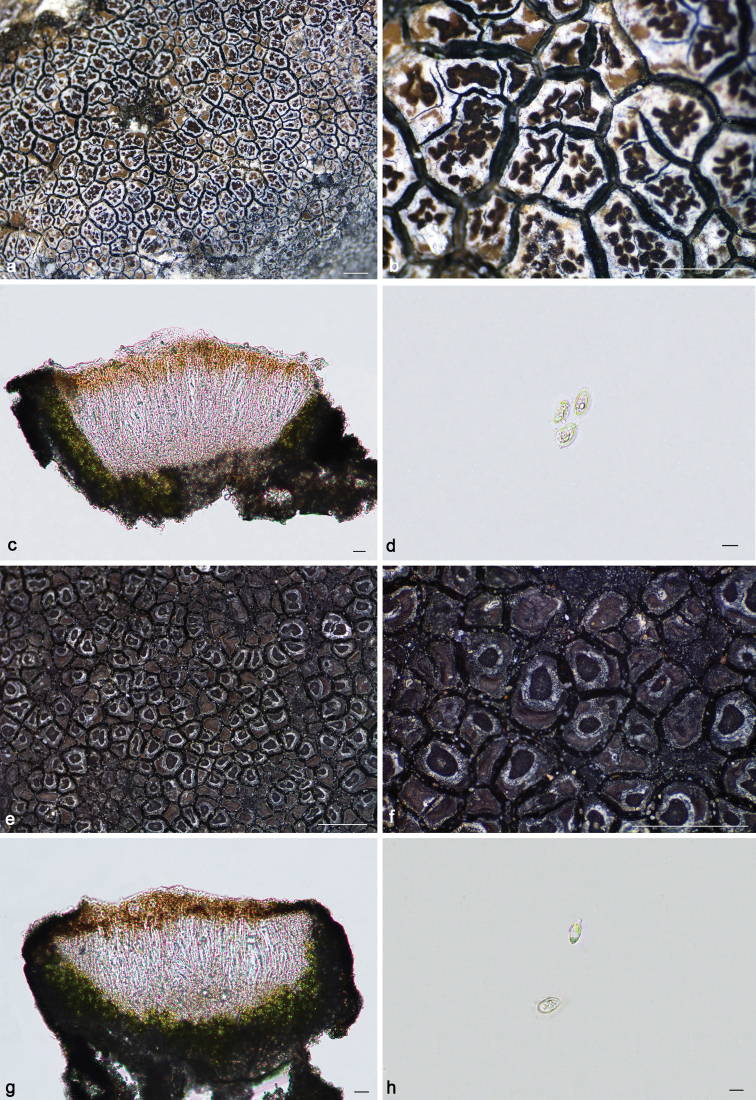
*Lecaimmeriabotryoides* (**a–d**KUN 20–66730): **a–b** thallus **c** apothecial anatomy. *L.lyagea* (**e–h**KUN 20–69070): **e–f** thallus **g** apothecial anatomy **h** scospores. Scale bars: 1 mm (**a–b, e–f**); 20 μm (**c, g**); 10 μm (**d, h**).

**Figure 8.**
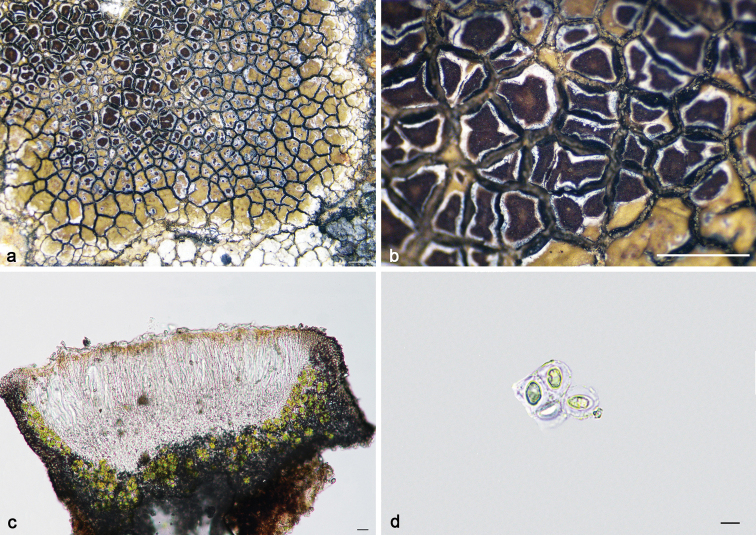
*Lecaimmeriamongolica* (**a–d** SDNU20190354): **a–b** thallus **c** apothecial anatomy **d** ascospores. Scale bars: 1 mm (**a–b**); 20 μm (**c**); 10 μm (**d**).

**Figure 9.**
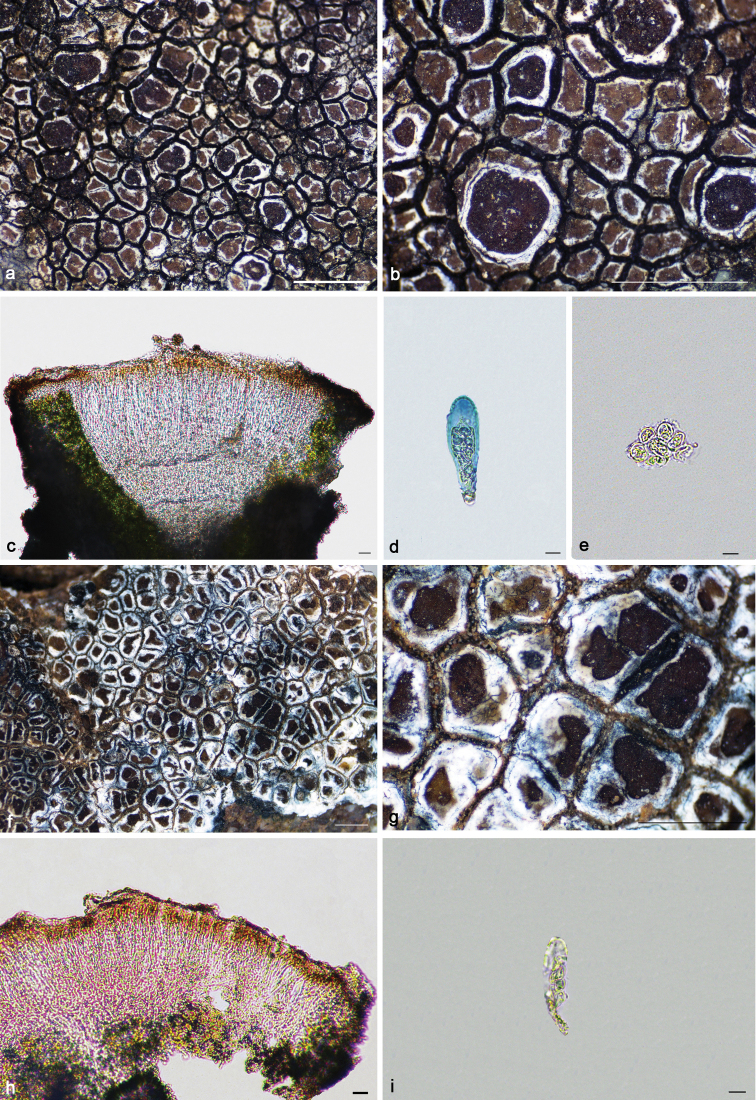
*Lecaimmeriaorbicularis* (**a–e**KUN 20–66753): **a–b** thallus **c** apothecial anatomy **d** ascus (Lugol’s iodine) **e** ascospores. *L.qinghaiensis* (**f–i**KUN 20–68696): **f–g** thallus **h** apothecial anatomy **i** ascus. Scale bars: 1 mm (**a–b, f–g**); 20 μm (**c, h**); 10 μm (**d, e, i**).

**Figure 10.**
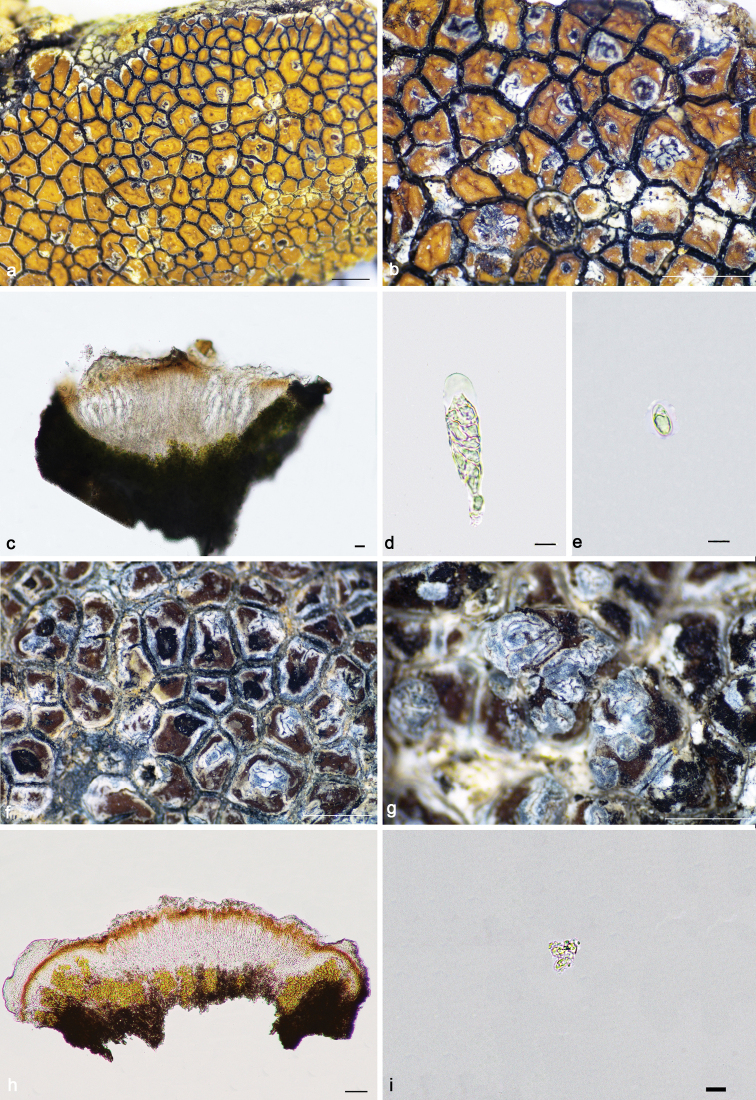
*Lecaimmeriatibetica* (**a–e**KUN XY19–1288): **a–b** thallus **c** apothecial anatomy **d** ascus **e** ascospore. *L.tuberculosa* (**f–i**KUN 18–58864): **f** thallus **g** conidiomata **h** apothecial anatomy **i** ascospores. Scale bars: 1 mm (**a–b, f–g**); 20 μm (**c**); 50 μm (**h**); 10 μm (**d, e, i**).

Thallus crustose, red-brown, orange-brown or dark brown, continuous or not; areolae irregular or tending to rectangular, with a glossy surface (*atrobrunnea*-type) caused by a layer of dead, colourless cells above the upper cortex; margin white or black; prothallus distinct at the margin of thallus or absent, sometimes developed between areolae. Upper cortex orange; epinecral layer colourless; algal layer continuous; medulla filled with grey granules. Apothecia lecanorine, immersed, round or irregular; disc red-brown, dark red-brown or dark orange-brown, flat or concave; margin present or absent, black or white, rarely green, pruinose or not. Exciple reduced, tissue at the lateral sides of the hymenium corresponding to the upper cortex and the algal layer of the vegetative areolae and to hypothecial hyphal cells when apothecia reach the margin of the areole (indicated in Figs 7c, g, 8c, 9c, h, 10c, h). Hymenium colourless; paraphyses simple, rarely branched, anastomosing or not; epihymeinum orange, orange-brown, rarely brown, with a plectenchyma. Asci *Porpidia*-type (indicated in Fig. 9d), cylindrical, eight-spored; ascospores ellipsoid, halonate, non-amyloid. Conidiomata present or absent, immersed, rarely convex, linear or stellate, rarely tuberculiform; conidia bacilliform.

#### Chemistry.

Thallus K–, C+/–. Medulla I+ violet. Gyrophoric acid, 4-O-demethylplanaic acid or no substances detected by TLC.

#### Ecology and distribution.

In China, growing on rock, sandstone, granite or Qilian jade (rare), from elevations of 3100 to 4800 m in the alpine zone of west China and from 1200 to 1900 m in the steppe of north China. This genus is known from China, Europe, Iran, Mongolia, Romania, Russia, and USA.

#### Notes.

The five-loci phylogenetic analysis showed that the species with lecanorine apothecia formed a novel lineage and should be excluded from *Immersaria*; thus, they are here treated as a new genus *Lecaimmeria*. *Lecaimmeria* is distinguished from related genera by its glossy surface, orange or red-brown areolae with margins, the amyloid medulla, the red-brown immersed lecanorine apothecia, the orange epihymenium with a plectenchyma and the *Porpidia*-type asci with eight halonate and non-amyloid ascospores. In China, the genus is distributed in alpine areas, high altitude desert-steppe areas or high latitude steppe. Almost all the species of *Lecaimmeria* grow on granite or sandstone, with the exception of one species, *L.tuberculosa*, which grows on jade. Interestingly, the margin of conidiomata and areolae of *Lecaimmeriatuberculosa* appear with heavily jade-green pruinose.

The immersed apothecia and brown thallus of *Lecaimmeria* often resemble those of *Immersaria*, but *Lecaimmeria* differs in its red-brown lecanorine apothecia, often with a white margin, their orange epihymenium with a plectenchyma and the thallus containing gyrophoric acid. This genus might be confused with *Bellemerea* by its lecanorine apothecia and the *Porpidia*-type asci with halonate ascospores, but the latter genus differs in its amyloid ascospores.

Three species, previously included in *Immersaria*, *I.cupreoatra*, *I.iranica* and *I.mehadiana*, have lecanorine apothecia, but two of these, *I.cupreoatra* and *I.mehadiana*, currently lack molecular sequences. We suggest that these three species should be transferred to *Lecaimmeria*, based on the following factors. Their morphology is consistent with *Lecaimmeria* according to molecular results and comparisons with type specimens, high-resolution photographs of the type materials and the original descriptions. One unknown “*Immersaria*” species from Macedonia is sister to *Lecaimmeriairanica* in the phylogenetic tree (Fig. 3), but comparison with high-resolution photograph and previous records (Malíček and Mayrhofer 2017) show that it differs in its black margin of areolae and absence of gyrophoric acid. This unknown species with lecanorine apothecia is possibly a member of *Lecaimmeria*, but descriptions are lacking and the specimens were not seen. Thus, this species is temporarily retained in *Immersaria*.

### ﻿ Lecaimmeria botryoides

Taxon classificationFungiLecidealesLecideaceae

C.M. Xie & Li S. Wang sp. nov.

F167841F-DFEB-5D2F-A674-9605842275B3

839744

Figure 7a–d

#### Etymology.

The name “botryoides” refers to the crowded apothecia while immature.

#### Type.

China. Sichuan Province: Aba City, Rangtang County, Haizi Mt., 4225 m elev., 32°21'N, 101°24'E, on rock, 6 Sept 2020, Li-Song Wang et al. 20–66730 (KUN-holotype).

#### Description.

Thallus areolate, red-brown, discontinuous; areolae 0.2–1.0 mm across, flat, slightly concave or convex, pruinose, polygonal, tending to be rectangular, margins heavily pruinose. Prothallus black, distinct in the margin of thallus. Upper cortex 20.0–25.0 μm thick, brown; epinecral layer 22.0–48.0 μm thick; algal layer ca. 37.0 μm thick, cells 7.5–10.0 μm diam., round. Apothecia frequent, irregular, densely crowded while immature (3–6/areolae), aggregate once mature, immersed, 0.2–1.3 mm in diam.; disc red-brown, flat, or concave, epruinose; margin pruinose, slightly raised. Hymenium 67.0–100.0 (–155.0) μm thick, colourless; paraphyses ca. 2.0 μm wide, simple, only branched at the top, not anastomosing; epihymenium 25.0–30.0 μm thick, orange; plectenchyma 2.0–8.0 μm thick; subhymenium 17.0–38.0 μm thick, colourless; hypothecium pale brown to brown. Asci *Porpidia*-type, cylindrical, eight-spored; ascospores 7.5–8.0 × 4.0–6.0 μm in diam., ellipsoid, halonate. Conidiomata not seen; conidia not seen.

#### Chemistry.

Thallus K–, C+/–. Medulla I+ violet. Chemotype Ⅰ: Gyrophoric acid. Chemotype Ⅱ: none.

#### Ecology and distribution.

In China, growing on rock at elevations of 3100–4300 m in the alpine zone. This species is known from Qinghai and Sichuan Provinces of China.

#### Notes.

*Lecaimmeriabotryoides* is characterised by its discontinuous thallus, densely crowded apothecia while immature and the orange epihymenium. *Lecaimmeriaorbicularis* is similar to *L.botryoides* in its red-brown thallus, but differs in its round, flat apothecia and continuous thallus. The red-brown thallus of *Lecaimmeriabotryoides* resembles *L.cupreoatra*, but the latter differs in the black margin of its apothecia and its distinct black prothallus between areolae.

#### Specimens examined (KUN).

China. Qinghai Province: Banma Co., 3958 m elev., 32°40'N, 100°48'E, on rock, 2020, Li-Song Wang et al. 20–66900, 3932 m elev., Li-Song Wang et al. 20–66898, 3935 m elev., Li-Song Wang et al. 20–66891, 3178 m elev., Li-Song Wang et al. 20–66765. Sichuan Province: Rangtang Co., Mt. Haizi, 4256 m elev., 32°21'N, 101°24'E, on rock, 2020, Li-Song Wang et al. 20–66721, 4300 m elev., Li-Song Wang et al. 20–67706, 4276 m elev., Li-Song Wang et al. 20–66706, 4255 m elev., Li-Song Wang et al. 20–66707, 4274 m elev., Li-Song Wang et al. 20–66713, 4274 m elev., Li-Song Wang et al. 20–66711, 20–66705, 4225 m elev., Li-Song Wang et al. 20–66730, 4220 m elev., 32°20'N, 101°25'E, Li-Song Wang et al. 20–66683.

### ﻿ Lecaimmeria cupreoatra

Taxon classificationFungiLecidealesLecideaceae

(Nyl.) C.M. Xie comb. nov.

1281AE93-E08C-5ADF-8589-22D011D47DF2

839745

#### Basionyms.

*Lecanoracupreoatra* Nyl., Lichens Lapponiae orientalis: 181 (1866).

#### Type.

Russia. “Medvæschiigora, ad Onegam”, 13 June 1863, Th. Simming, H9508237 (H-lectotype!).

#### Description.

Nylander (1866) and Clauzade and Roux (1985).

#### Notes.

The lectotype grows on siliceous rock and contains several intact apothecia. As “*Immersaria*” *cupreoatra* has lecanorine apothecia and is related to *I.lygeae* in our phylogeny, it is, therefore, transferred to *Lecaimmeria*. This species has not been correctly recorded in China (see notes for *Lecaimmeriamongolica*). The species is known from Europe, Mongolia, Russia, and USA (Calatayud and Rambold 1998).

#### Specimens examined (H).

Russia. Kl. Kurkijoki, Kuuppala, Himohirsi, 12 May 1934, Räsänen, V., H9503417, H9510194.

### ﻿ Lecaimmeria iranica

Taxon classificationFungiLecidealesLecideaceae

(Valadb., Sipman & Rambold) C.M. Xie comb. nov.

6DA2EA0E-439D-5DBE-895D-A831F71661DC

839746

#### Basionyms.

*Immersariairanica* Valadb., Sipman & Rambold, Lichenologist 43(3): 204 (2011).

#### Type.

Iran. Mazandaran, Haraz Road, 20 km to Aamol, 36°17'N, 52°21'E, on calcareous rock, 1475 m, 7 Apr 2006, T. Valadbeigi 9008 (TARI-holotype; B, hb. Valadbeigi-isotype). Not seen.

#### Description.

Valadbeigi et al. (2011).

#### Notes.

“*Immersaria*” *iranica* has lecanorine apothecia, a distinct epinecral layer and halonate ascospores (Valadbeigi et al. 2011). The materials from China are in accordance with the materials of Iran, based on comparisons with the original descriptions and the photographs given by Valadbeigi et al. (2011). The characters of this species are consistent with the new genus and the phylogenetic results showed that it was clustered with species of *Lecaimmeria*. Therefore, it was transferred to *Lecaimmeria*. This species is currently known from Iran and China.

#### Specimens examined (SDNU).

China. Xinjiang: Urumqi, Mt. Tianshan-glacier No.1, alt. 3800 m, on rock, 2011, Z.L. Huang SDNU20126106, SDNU20129049.

### ﻿ Lecaimmeria lygaea

Taxon classificationFungiLecidealesLecideaceae

C.M. Xie & Lu L. Zhang sp. nov.

55D6DE48-A23E-580E-9D35-37E7D198288E

839747

Figure 7e–h

#### Etymology.

The name “lygaea” refers to the dark appearance of the thallus.

#### Type.

China. Tibet: Changdu City, Mangkang County, Luoni Village, 4127 m elev., 29°56'N, 98°33'E, on rock, 24 Sept 2020, Li-Song Wang et al. 20–69072 (KUN-holotype).

#### Description.

Thallus areolate, dark red-brown, dark brown, continuous; areolae 0.5–1.0 mm across, flat, epruinose, irregular pentagonal, sometimes rectangular, fissures between areolae often filled with black prothallus; margin black, thinly pruinose; prothallus black, developed between areolae, also distinct in the margin. Upper cortex ca. 20.0 μm thick, orange-brown; epinecral layer ca. 15.0 μm thick; algal layer ca. 50.0 μm thick, cells 7.0–13.0 μm in diam., round. Apothecia frequent, round, crowded, immersed, 0.2–0.8 mm in diam.; disc red-brown, flat, or concave, epruinose; margin black, moderately thick, pruinose, raised. Hymenium 75.0–93.0 μm thick, colourless; paraphyses ca. 2.0 μm wide, simple, unbranched, not anastomosing; epihymenium 25.0–38.0 μm thick, orange brown; plectenchyma ca. 7.0 μm thick; subhymenium 20.0–25.0 μm thick, colourless; hypothecium brown Asci *Porpidia*-type, cylindrical, eight-spored; ascospores 12.5–20.0 × 5.0–7.5 μm in diam., ellipsoid, halonate. Conidiomata immersed, stellate, black, margin pruinose; conidia 5.0 × 1.0 μm, bacilliform.

#### Chemistry.

Thallus K–, C–. Medulla I+ violet. Unknown fatty acid by TLC.

#### Ecology and distribution.

In China, growing on sandstone at elevations of 4000–4200 m in the alpine zone. This species is known from the Tibet Region of China.

#### Notes.

*Lecaimmerialygaea* is characterised by its dark brown thallus, black margin of its areolae, black prothallus which fills the fissures between areolae, dark orange apothecia and its orange brown epihymenium. *Lecaimmeriacupreoatra* and *L.mehadiana* are similar to *L.lygaea*, but *L.cupreoatra* has a discontinuous thallus, with each areola surrounded by black prothallus, dark red-brown to black-brown apothecia without a margin. *Lecaimmeriamehadiana* has areolae with a white margin, black-brown apothecia, brown epihymenium and contains 4-O-demethylplanaic acid. The phylogenetic results show that *Lecaimmeriatibetica* is the sister species to *L.lygaea.* They are similar in chemistry, but differ in its orange-brown thallus and dark orange brown apothecia.

#### Specimens examined (KUN).

China. Tibet: Changdu City, Mangkang Co., Luoni Village, 4099 m elev., 29°56'N, 98°33'E, on rock, 2020, Li-Song Wang et al. 20–69054, 4131 m elev., Li-Song Wang et al. 20–69070, 4127 m elev., Li-Song Wang et al. 20–69072, 4095 m elev., Li-Song Wang et al.20–69053.

### ﻿ Lecaimmeria mehadiana

Taxon classificationFungiLecidealesLecideaceae

(Calatayud & Rambold) C.M. Xie comb. nov.

83DCC356-A956-5F4D-AA4E-B446B600347B

839748

#### Basionyms.

*Immersariamehadiana* Calat & Rambold, Lichenologist 30(3): 233 (1998).

#### Type.

Romania. Caras-Severin Comitat, Mehadía, Strájot Mtn., on rock, 1994, Rambold, G.W., M0101781 (M-holotype!), M0101780, M0101782, M0101783 (M-isotype!). High-resolution photographs seen.

#### Description.

Calatayud and Rambold (1998).

#### Notes.

As “*Immersaria*” *mehadiana* has lecanorine apothecia and resembles *L.lygaea* and *L.cupreoatra*, having a dark brown thallus, it is, therefore, transferred to *Lecaimmeria*. This species is characterised by its greyish prothallus, dark brown apothecia and the brown epihymenium. It is only known from Romania (Calatayud and Rambold 1998).

### ﻿ Lecaimmeria mongolica

Taxon classificationFungiLecidealesLecideaceae

C.M. Xie & Lu L. Zhang sp. nov.

0B67465A-4C62-5C9C-A1C3-8434914B523E

839749

Figure 8a–d

#### Etymology.

The name “mongolica” refers to the collection of the holotype within Inner Mongolia, an autonomous region of China.

#### Type.

China. Inner Mongolia: Chifeng City, Balinyouqi, Han Mountain, 1445m elev., 44°11'N, 118°44'E, on rock, 22 Jul 2019, Zun-Tian Zhao et al. SDNU20190354 (SDNU-holotype).

#### Description.

Thallus areolate, orange, continuous; areolae 0.4–0.8 mm across, epruinose, neatly arranged, irregular, tending to be rectangular, margin pruinose; prothallus black, not distinct. Upper cortex ca. 20.0 μm thick, brown; epinecral layer 5.0–8.0 μm thick; algal layer ca. 87.0 μm thick, cells 7.5–12.5 μm diam., round. Apothecia frequent, crowded, immersed or isolated from areolae, 0.2–0.8 mm in diam.; disc red-brown, flat, slightly convex, epruinose; margin pruinose. Hymenium 62.0–83.0 μm thick, colourless; paraphyses ca. 2.0 μm wide, unbranched, not anastomosing; epihymenium ca. 42.0 μm thick, orange; plectenchyma 5.0–10.0 μm thick; subhymenium 30.0–38.0 μm thick, colourless; hypothecium brown. Asci *Porpidia*-type, cylindrical, eight-spored; ascospores 10.0–17.5 × 6.0–7.5 μm in diam., ellipsoid, halonate. Conidiomata immersed, oblate, rare ellipsoid, black, margin pruinose; conidia 5.0 × 1.0 μm, bacilliform.

#### Chemistry.

Thallus K–, C+. Medulla I+ violet. Gyrophoric acid.

#### Ecology and distribution.

In China, growing on granite at elevations of 1400–2000 m in steppe or mountains. This species is known from Inner Mongolia of China.

#### Notes.

This species was once reported as “*Immersaria*” *cupreoatra* from China (Zhang et al. 2015), but after comparing our collections with the type material, this was found to be a misclassification. Additionally, the phylogenetic results showed that these collections formed a well-supported lineage belonging to *Lecaimmeria*. Therefore, it is here treated as a new species, *Lecaimmeriamongolica*, characterised by its orange-brown thallus, red-brown apothecia with a distinct white margin and the thallus containing gyrophoric acid. *Lecaimmeriacupreoatra* resembles *L.mongolica* by containing gyrophoric acid, but it differs in its dark black brown thallus and the black margin of its apothecia. *Lecaimmeriatibetica* is similar to *L.mongolica* in its orange thallus, but differs in its smaller, dark orange apothecia and that no substance can be detected by TLC.

#### Specimens examined (SDNU).

China. Inner Mongolia: Chifeng City, Balin Youqi, Mt. Qingyangcheng, 1445 m elev., 43°35'N, 117°30'E, on rock, 2019, Zun-Tian Zhao et al. SDNU20190350; Han Shan, 1563 m elev., 44°11'N, 118°44'E, on rock, Zun-Tian Zhao et al. SDNU20190354; A’ershan City, Mt. Jiguan, 1500 m elev., on rock, 2011, Yu-Liang Cheng SDNU20124912, 1400 m elev., Dai-Feng Jiang SDNU20124859; Ke Qi, Huanggangliang, 2000 m elev., on rock, Pan-Meng Wang SDNU20117613, Xing-Ran Kou SDNU20117399.

### ﻿ Lecaimmeria orbicularis

Taxon classificationFungiLecidealesLecideaceae

C.M. Xie & Lu L. Zhang sp. nov.

8DD23394-FDE8-5B5E-87EA-E58EF75F5627

839750

Figure 9a–e

#### Etymology.

The name “orbicularis” refers to the round shape of the apothecia.

#### Type.

China. Sichuan Province: Rangtang Co., Gangmuda Village, 3800 m elev., 32°18'N, 101°3'E, on rock, 7 Sept 2020, Li-Song Wang et al. 20–66753 (KUN-holotype).

#### Description.

Thallus areolate, red-brown, rarely orange-brown, continuous; areolae 0.2–1.0 mm across, flat, occasionally wrinkled, tending to rectangular, fissures between areolae often filled with black prothallus, margin pruinose; prothallus black, developed between areolae, also distinct in the margin. Upper cortex 42.0–58.0 μm thick, brown; epinecral layer 5.0–20.0 μm thick; algal layer 70.0–113.0 μm thick, cells 10.0–15.0 × 7.5–10.0 μm in diam., ellipsoid to round. Apothecia frequent, scattered, immersed or isolated from areolae, 0.5–1.3 mm in diam.; disc red-brown, flat, round, epruinose; margin white, slightly raised. Hymenium 75.0–113.0 μm thick, colourless; paraphyses ca. 2.0 μm wide, simple, unbranched, not anastomosing; epihymenium 17.5–30.0 μm thick, orange; plectenchyma 5.0–15.0 μm thick; subhymenium 30.0–63.0 μm thick, colourless; hypothecium brown. Asci *Porpidia*-type, cylindrical, eight-spored; ascospores 12.5–15.0 × 5.0–6.0 μm, ellipsoid, halonate. Conidiomata not seen.

#### Chemistry.

Thallus K–, C–. Medulla I+ violet. None.

#### Ecology and distribution.

In China, growing on granite or sandstone at elevations of 3700–4200 m in the alpine zone. This species is known from Qinghai and Sichuan Provinces of China.

#### Notes.

*Lecaimmeriaorbicularis* is characterised by its orange brown thallus, neatly arranged areolae and round, flat apothecia. *Lecaimmeriabotryoides* is similar to *L.orbicularis* (see notes for *L.botryoides*). *Lecaimmeriamongolica* might be confused with *L.orbicularis* due to its large apothecia with a white margin, but differs in its red-brown thallus and distribution in steppes. The red-brown thallus of *Lecaimmeriacupreoatra* resembles that of *L.orbicularis*, but differs in the black margin of its apothecia and its distinct black prothallus between areolae.

#### Specimens examined (KUN).

China. Qinghai Province: Jiuzhi Co., Nianbaoyuze, 4200 m elev., 33°14'N, 100°58'E, on rock, 2020, Li-Song Wang et al. 20–66811, 20–66829, 20–66801, 20–66826A, 20–66821, 20–66805, 20–66833, 20–66817, 20–66841; Banma Co., Nianbaoyuze, 3930 m elev., 32°40'N, 100°48'E, Li-Song Wang et al. 20–66909, 20–66908, 20–66896, 20–66886B, 20–66899, 20–66935, 20–66943; Zhiqingsongduo Town, 3712 m elev., 33°24'N, 101°25'E, Li-Song Wang et al. 20–66965; Suohurima Village, 4029 m elev., 33°23'N, 100°57'E, Li-Song Wang et al. 20–66979. Sichuan Province: Rangtang Co., Gangmuda Village, 3800 m elev., 32°18'N, 101°3'E, on rock, 2020, Li-Song Wang et al. 20–66753, 20–66750, 3793 m elev., Li-Song Wang et al. 20–66747, Shangrangtang Village, 3730 m elev., 32°16'N, 101°21'E, Li-Song Wang et al. 20–66743.

### ﻿ Lecaimmeria qinghaiensis

Taxon classificationFungiLecidealesLecideaceae

C.M. Xie & Li S. Wang sp. nov.

2F83DD1A-909C-525F-A943-23DEF53F1C97

839751

Figure 9f–i

#### Etymology.

The name “qinghaiensis” refers to the location in which the holotype was collected, in “Qinghai”, a province of China.

#### Type.

China. Qinghai Province: Yushu City, Zaduo County, Sahuteng Town, 4634 m elev., 32°55'N, 95°46'E, on rock, 20 Sept 2020, Li-Song Wang et al. 20–68698 (KUN-holotype).

#### Description.

Thallus areolate, yellow-brown, rusty, continuous; areolae 0.5–1.5 mm across, flat, epruinose; margin pruinose, occasionally green pigmented; prothallus black, distinct at the margin. Upper cortex 27.0–38.0 μm thick, brown; epinecral layer 12.0–20.0 μm thick; algal layer 57.0–93.0 μm thick, cells 7.5–12.5 × 5.0–12.5 μm in diam., ellipsoid to round. Apothecia frequent, immersed or isolated from areolae, round or somewhat irregular while immature, ellipsoid and tending to be rectangular or occupying the whole areolae once mature, 0.2–1.3 mm in diam.; disc brown, dark red-brown, flat, occasionally with a fissure when mature, epruinose; margin white, slightly raised. Hymenium 52.0–63.0 μm thick, colourless; paraphyses 2.0–3.0 μm wide, unbranched, not anastomosing; epihymenium 25.0–30.0 μm thick, dark orange-brown; plectenchyma 7.0–18.0 μm thick; subhymenium 50.0–63.0 μm thick, colourless; hypothecium brown. Asci *Porpidia*-type, cylindrical, eight-spored; ascospores 8.0–15.0 × 5.0–7.5 μm in diam., ellipsoid, not distinctly halonate. Conidiomata rare, immersed, flat, slightly convex, liner, stellate, graphidoid once mature, black, margin pruinose; conidia not seen.

#### Chemistry.

Thallus K–, C–. Medulla I+ violet. None.

#### Ecology and distribution.

In China, growing on rock at elevations of 4600–4900 m in the alpine zone. This species is known from Qinghai Province of China.

#### Notes.

*Lecaimmeriaqinghaiensis* is characterised by the yellow-brown, rusty thallus, the red-brown apothecia often occupying the whole areolae at maturity and the dark orange-brown epihymenium. The phylogenetic results showed that *Lecaimmeriatuberculosa* is the sister species to *L.qinghaiensis* which is similar in the appearance of the thallus, but differs in the brown, never rusty thallus, the red-brown apothecia and the green, tuberculiform conidiomata. The red-brown apothecia of *Lecaimmeriaqinghaiensis* resembles *L.iranica*, but differs in the rusty thallus and the white margin of the apothecia.

#### Specimens examined (KUN).

China. Qinghai Province: Zaduo Co., Sahuteng Town, 4634 m elev., 32°55'N, 95°46'E, on rock, 2020, Li-Song Wang et al. 20–68698, 4637 m elev., Li-Song Wang et al. 20–68687, 4622 m elev., Li-Song Wang et al. 20–68696; 4790 m elev., 33°31'N, 95°8'E, Xin-Yu Wang et al. 20–3115, 4791 m elev., Xin-Yu Wang et al. 20–3127; Zaqing Village, 4815 m elev., Xin-Yu Wang et al. 20–849.

### ﻿ Lecaimmeria tibetica

Taxon classificationFungiLecidealesLecideaceae

C.M. Xie & Xin Y. Wang sp. nov.

FB7AB44C-9DB5-5FD6-A719-CA5DD3B56B16

839752

Figure 10a–e

#### Etymology.

The name “tibetica” refers to the location from which the holotype was collected: “Tibet”, an autonomous region of China.

#### Type.

China. Tibet: Gongga Co., Jiangtang Town, 4557 m elev., 29°12'N, 90°38'E, on rock, 9 Sept 2019, Xin-Yu Wang et al. XY19–1291 (KUN-holotype).

#### Description.

Thallus areolate, orange-brown, epruinose; areolae 0.3–0.5 mm across, irregular, upper surface uneven, margin lacking, pruinose; prothallus black, distinct at the margin. Upper cortex 17.0–33.0 μm thick, brown; epinecral layer 10.0–20.0 μm thick; algal layer ca. 75.0 μm thick, cells 7.0–10.0 diam., round. Apothecia rare, immersed or isolated from areolae, 0.2–0.5 mm in diam.; disc dark orange-brown, epruinose, flat, slightly convex; margin pruinose. Hymenium 105.0–138.0 μm thick, colourless; paraphyses ca. 2.0 μm wide, unbranched, not anastomosing; epihymenium ca. 25.0 μm thick, orange; plectenchyma ca. 12.0 μm thick; subhymenium almost absent, colourless; hypothecium brown. Asci *Porpidia*-type, cylindrical, eight-spored; ascospores 12.5–15.0 × 5.0–6.0 μm, ellipsoid, halonate. Conidiomata immersed, oblate, black, margin pruinose; conidia 5.0 × 1.5–2.0 μm in diam., bacilliform.

#### Chemistry.

Thallus K–, C–. Medulla I+ violet. None.

#### Ecology and distribution.

In China, growing on quartz sandstone at elevations of 4300–4600 m in the alpine zone. This species is known from Tibet, China.

#### Notes.

*Lecaimmeriatibetica* is characterised by the orange-brown thallus, the black pigmentation of the areolae margin and the dark orange-brown and small size of the apothecia. *Lecaimmeriatibetica* is similar to *L.mongolica* (see notes for *L.mongolica*). The red-brown apothecia of *Lecaimmeriacupreoatra* resembles *L.tibetica*, but that species differs in its dark red-brown thallus and the presence of gyrophoric acid.

#### Specimens examined (KUN).

China. Tibet: Gongga Co., Jiangtang Town, 4583 m elev., 29°12'N, 90°38'E, on rock, 2019, Xin-Yu Wang et al. XY19–1288, 4557 m elev., XY19–1291, 4560 m elev., XY19–1280; Dingri Co., Zhaguozhong, 4310 m elev., 28°35'N, 86°53'E, Li-Song Wang et al. 19–64071.

### ﻿ Lecaimmeria tuberculosa

Taxon classificationFungiLecidealesLecideaceae

C.M. Xie & Xin Y. Wang sp. nov.

8C954684-2827-5F1D-8583-3FB93FE9904F

839754

Figure 10f–i

#### Etymology.

The name “tuberculosa” refers to the tuberculiform conidiomata.

#### Type.

China. Gansu Province: Zhangye City, Sunan Co., Along the way from Sunan to Qilian, 3928 m elev., 38°37'N, 99°28'E, on rock, 30 May 2018, Li-Song Wang et al. 18–58865 (KUN-holotype).

#### Description.

Thallus areolate, red-brown, continuous; areolae 0.5–1.3 mm across, slightly convex, epruinose; margin pruinose, often jade-green pigmented; prothallus not distinct. Upper cortex ca. 27.0 μm thick, orange; epinecral layer up to 28.0 μm thick, uneven, sometimes absent; algal layer ca. 50.0 μm thick, cells 6.0–10.0 μm diam., round. Apothecia frequent, scattered, immersed, 0.3–0.6 mm in diam.; disc red-brown, concave, epruinose; margin absent. Hymenium 55.0–83.0 μm thick, colourless; paraphyses ca. 2.0 μm wide, unbranched, not anastomosing; epihymenium 15.0–30.0 μm thick, orange; plectenchyma ca. 5.0 μm thick, discontinuous; subhymenium ca. 38.0 μm thick, colourless; hypothecium brown. Asci *Porpidia*-type, cylindrical, eight-spored; ascospores 6.0–12.5 × 3.0–5.0 μm in diam., ellipsoid, halonate. Conidiomata stellate, strongly convex, rarely immersed, forming tuberculiform, black, margin pruinose, jade-green pigmented; conidia 3.0–4.5 × 1.0 μm in diam., oblong to bacilliform.

#### Chemistry.

Thallus K–, C–. Medulla I+ violet. No substances by TLC.

#### Ecology and distribution.

In China, growing on the Qilian jade or sandstone at elevations of 3900–4200 m in the alpine zone. This species is known from Qinghai Province and Gansu Province of China.

#### Notes.

*Lecaimmeriatuberculosa* is characterised by its red-brown thallus, the jade-green pruinose colour at the margin of its areolae, its red-brown, concave apothecia without a proper margin and tuberculiform conidiomata. *Lecaimmeriaqinghaiensis* is similar to *L.tuberculosa* (see notes for *L.qinghaiensis*). *Lecaimmeriatuberculosa* usually grows on jade and, interestingly, the margin of the conidiomata and areolae of the species are heavily jade-green pigmented. *Lecaimmeriairanica* resembles *L.tuberculosa* by the absence of an apothecial margin, but differs in its immersed conidiomata and the white margin of its areolae.

#### Specimens examined (KUN).

China. Qinghai Province: Gande Co., Qingzhen Village, 4124 m elev., 34°11'N, 100°12'E, on rock, 2020, Li-Song Wang et al. 20–68077, 4145 m elev., Li-Song Wang et al. 20–68055. Gansu Province: Zhangye City, Sunan Co., along the way from Sunan to Qilian, 3928 m elev., 38°37'N, 99°28'E, on rock, 2018, Li-Song Wang et al. 18–58856, 18–58857, 18–58865, 18–59835.

### ﻿Key to species of *Immersaria* in China

**Table:** 

1	Thallus greyish-brown; apothecia crowded	***I.ferruginea***
–	Thallus reddish-brown; apothecia rarely crowded	**2**
2	Thallus orange; areolae irregular	***I.aurantia***
–	Thallus not orange; areolae irregular, polygonal or rectangular	**3**
3	Thallus large, 6–10 cm across; areolae aggregated by several smaller areolae and black prothallus	***I.shangrilaensis***
–	Thallus smaller, 2–5 cm across; areolae not aggregated	**4**
4	Thallus areolae convex, not rusty, not cracked; ascospores over 15 μm long	***I.athroocarpa***
–	Thallus areolae flat, often rusty, cracked; ascospores never over 15 μm long	***I.venusta***

### ﻿Key to species of *Lecaimmeria* in China

**Table:** 

1	Prothallus distinct and filling the fissures between areolae	***L.lygaea***
–	Prothallus only distinct at the margin	**2**
2	Thallus orange	**3**
–	Thallus reddish-brown	**4**
3	Apothecia red-brown; containing gyrophoric acid	***L.mongolica***
–	Apothecia dark orange; no substance detected by TLC	***L.tibetica***
4	Apothecia margin absent	**5**
–	Apothecia margin present	**6**
5	Areolae margin white; epihymenium brown; pycindia immersed, linear or stellate	***L.iranica***
–	Areolae margin green; epihymenium orange; pycindia convex, tuberculiform	***L.tuberculosa***
6	Thallus rusty; apothecia occupy the whole areolae	***L.qinghaiensis***
–	Thallus not rusty; apothecia do not occupy the areolae	**7**
7	Apothecia irregular, crowded while immature, aggregate when mature	***L.botryoides***
–	Apothecia round, rarely crowded, not aggregate	***L.orbicularis***

## Supplementary Material

XML Treatment for Immersaria

XML Treatment for Immersaria athroocarpa

XML Treatment for Immersaria aurantia

XML Treatment for Immersaria ferruginea

XML Treatment for Immersaria shangrilaensis

XML Treatment for Immersaria venusta

XML Treatment for Lecaimmeria

XML Treatment for Lecaimmeria botryoides

XML Treatment for Lecaimmeria cupreoatra

XML Treatment for Lecaimmeria iranica

XML Treatment for Lecaimmeria lygaea

XML Treatment for Lecaimmeria mehadiana

XML Treatment for Lecaimmeria mongolica

XML Treatment for Lecaimmeria orbicularis

XML Treatment for Lecaimmeria qinghaiensis

XML Treatment for Lecaimmeria tibetica

XML Treatment for Lecaimmeria tuberculosa

## Supplementary materials

Supplementary material 1 Table S1 Data type docx. file Explanation note A list of specimens and GenBank accession number of sequences used in this study. The new sequences generated are in bold. https://binary.pensoft.net/file/646464This dataset is made available under the Open Database License (http://opendatacommons.org/licenses/odbl/1.0/). The Open Database License (ODbL) is a license agreement intended to allow users to freely share, modify, and use this Dataset while maintaining this same freedom for others, provided that the original source and author(s) are credited.Cong-Miao Xie, Li-Song Wang, Zun-Tian Zhao, Yan-Yun Zhang, Xin-Yu Wang, Lu-Lu Zhang
