# Supplementary material for: ﻿Revision of Immersaria and a new lecanorine genus in Lecideaceae (lichenised Ascomycota, Lecanoromycetes)
Source: MycoKeys. 2022 Feb 15;87:99–132. doi: 10.3897/mycokeys.87.72614 (PMC8863769; doi:10.3897/mycokeys.87.72614)
Supplement: Supplementary material 1 — Table S1 [file mycokeys-87-099-s001.doc]

**Supplementary Table 1.** A list of specimens and GenBank accession number of sequences used in this study. The new sequences generated are in bold.

| **Taxa** | **Locality** | **Voucher Specimens** | **GenBank accession number** | | | | |
| --- | --- | --- | --- | --- | --- | --- | --- |
|  |  |  | **nrITS** | **nrLSU** | **RPB1** | **RPB2** | **mtSSU** |
| *Amygdalaria consentiens* | Greenland | J. Buschbom 22.8.2000-56 | — | AY532977 | — | — | — |
| *A. elegantior* | Canada | J. Buschbom 16.7.2000-38 | — | AY532979 | — | — | — |
| *A. panaeola* | USA | J. Buschbom 3118 | — | AY532980 | — | — | — |
| *Bellemerea alpina* | Unknown | Hafellner 46531 | AF332117 | — | — | — | — |
| *B. alpina* | Greenland | J. Buschbom 23.8.2000-22 | — | AY532982 | — | — | — |
| *B. cinereorufescens* | USA | McCune 35490 | KY800500 | — | — | — | — |
| *B. subsorediza* | Sweden | J. Buschbom 25.8.2001-38a | — | AY532983 | — | — | — |
| *Bryobilimbia australis* | Chile | S. Pérez-Ortega 1419 | KF683092 | — | KF683112 | — | — |
| *B. diapensiae* | Sweden | Arup L04400 | HQ650660 | HQ660539 | — | — | HQ660564 |
| *B. hypnorum* | Unknown | F. Jonsson & Z. Palice 10747 | KF683093 | — | — | — | — |
| *B. sanguineoatra* | France | Sipman 44029 | HQ650664 | AY533005 | — | — | — |
| *Clauzadea monticola* | USA | A. M. Fryday 9703 | KF683090 | KF692710 | — | — | — |
| *C. monticola* | USA | A. M. Fryday 9728 | — | — | KF683113 | KF683110 | — |
| *Cyclohymenia epilithica* | USA | McCune 31029 | KY800503 | KY800514 | — | — | — |
| *C. epilithica* | USA | McCune 31068 | KY800504 | KY800513 | — | — | — |
| *Farnoldia jurana* | Austria | Herbarium Tuerk 39660 | EU263920 | — | MK684889 | — | GU074511 |
| *F. jurana* | Unknown | J. Buschbom 15.09.2001-1 | — | AY532984 | — | — | — |
| ***Immersaria athroocarpa*** | **China: Inner** Mongolia | **SDNU20190035** | **MZ227390** | **MZ227079** | **—** | **MZ343462** | **MZ227025** |
| ***I. athroocarpa*** | **China: Inner** Mongolia | **SDNU20190140** | **MZ227391** | **MZ227080** | **MZ343432** | **MZ343461** | **MZ227026** |
| ***I. athroocarpa*** | **China: Inner** Mongolia | **SDNU20190143** | **MZ227393** | **MZ227081** | **MZ343439** | **—** | **—** |
| ***I. athroocarpa*** | **China: Inner** Mongolia | **SDNU20190227D** | **MZ227394** | **—** | **MZ343433** | **—** | **—** |
| ***I. athroocarpa*** | **China: Inner** Mongolia | **SDNU20190227** | **MZ227395** | **—** | **MZ343434** | **—** | **—** |
| ***I. aurantia*** | **China: Qinghai** | **KUN 20-67809** | **MZ227428** | **MZ227101** | **MZ343431** | **—** | **MZ227031** |
| ***I. aurantia*** | **China: Sichuan** | **KUN 20-66701** | **MZ227400** | **MZ227092** | **—** | **—** | **—** |
| ***I. aurantia*** | **China: Sichuan** | **KUN 20-66708A** | **MZ227403** | **MZ227093** | **MZ343427** | **—** | **—** |
| ***I. aurantia*** | **China: Sichuan** | **KUN 20-66680** | **MZ227483** | **MZ227087** | **—** | **—** | **—** |
| ***I. aurantia*** | **China: Sichuan** | **KUN 20-66687** | **MZ227485** | **MZ227088** | **—** | **—** | **—** |
| ***I. aurantia*** | **China: Sichuan** | **KUN 20-66692** | **MZ227486** | **MZ227089** | **—** | **—** | **—** |
| ***I. aurantia*** | **China: Sichuan** | **KUN 20-66693** | **MZ227487** | **MZ227090** | **—** | **—** | **—** |
| ***I. ferruginea*** | **China: Sichuan** | **KUN 20-66697A** | **MZ227399** | **MZ227091** | **—** | **—** | **—** |
| ***I. ferruginea*** | **China: Sichuan** | **KUN 20-67670** | **MZ227426** | **MZ227100** | **MZ343441** | **—** | **—** |
| ***I. ferruginea*** | **China: Tibet** | **KUN 20-69105** | **MZ227463** | **MZ227115** | **—** | **MZ343469** | **—** |
| ***I. ferruginea*** | **China: Tibet** | **KUN 20-69144** | **MZ227466** | **MZ227118** | **MZ343417** | **MZ343470** | **MZ227034** |
| ***I. ferruginea*** | **China: Tibet** | **KUN 20-69146** | **MZ227467** | **MZ227119** | **—** | **MZ343471** | **—** |
| ***I. ferruginea*** | **China: Tibet** | **KUN 20-69148** | **MZ227468** | **MZ227120** | **—** | **MZ343468** | **—** |
| ***I. shangrilaensis*** | **China: Yunnan** | **SDNU20181696** | **MZ227392** | **—** | **—** | **—** | **—** |
| ***I. shangrilaensis*** | **China: Yunnan** | **KUN 18-60447** | **MZ227472** | **MZ227083** | **—** | **—** | **—** |
| ***I. venusta*** | **China: Qinghai** | **KUN 20-66933** | **MZ227439** | **MZ227105** | **MZ343438** | **—** | **—** |
| ***I. venusta*** | **China: Qinghai** | **KUN 20-66940** | **MZ227441** | **MZ227106** | **MZ343406** | **—** | **—** |
| ***I. venusta*** | **China: Qinghai** | **KUN 20-68157** | **MZ227452** | **MZ227112** | **MZ343424** | **MZ343458** | **—** |
| ***I. venusta*** | **China: Qinghai** | **KUN 20-66796** | **MZ227413** | **MZ227097** | **MZ343430** | **—** | **—** |
| ***I. venusta*** | **China: Qinghai** | **KUN 20-66808** | **MZ227416** | **MZ227098** | **MZ343426** | **—** | **MZ227028** |
| ***I. venusta*** | **China: Qinghai** | **KUN 20-66810** | **MZ227417** | **MZ227099** | **MZ343428** | **MZ343473** | **MZ227029** |
| ***I. venusta*** | **China: Qinghai** | **KUN 20-66824** | **MZ227421** | **—** | **MZ343423** | **MZ343460** | **—** |
| ***I. venusta*** | **China: Qinghai** | **KUN 20-66977** | **MZ227444** | **MZ227107** | **—** | **MZ343466** | **—** |
| ***I. venusta*** | **China: Qinghai** | **KUN 20-2811** | **MZ227480** | **MZ227085** | **MZ343440** | **—** | **—** |
| ***I. venusta*** | **China: Qinghai** | **KUN 20-2799** | **MZ227481** | **—** | **MZ343398** | **—** | **—** |
| ***I. venusta*** | **China: Qinghai** | **KUN 20-67959** | **MZ227447** | **MZ227109** | **MZ343437** | **—** | **—** |
| ***I. venusta*** | **China: Qinghai** | **KUN 20-67965** | **MZ227448** | **MZ227110** | **MZ343429** | **—** | **—** |
| ***I. venusta*** | **China: Qinghai** | **KUN 20-67969A** | **MZ227449** | **MZ227111** | **MZ343425** | **—** | **—** |
| ***I. venusta*** | **China: Sichuan** | **KUN 20-66607** | **MZ227482** | **MZ227086** | **—** | **—** | **—** |
| ***I. venusta*** | **China: Sichuan** | **KUN 20-66721C** | **MZ227407** | **MZ227094** | **—** | **—** | **—** |
| ***I. venusta*** | **China: Sichuan** | **KUN 20-66725** | **MZ227409** | **MZ227096** | **—** | **—** | **—** |
| ***I. venusta*** | **China: Sichuan** | **KUN 20-68802** | **MZ227456** | **MZ227113** | **MZ343399** | **MZ343459** | **—** |
| *Immersaria* sp. | Macedonia | Malicek 7717 | MF149862 | — | — | — | MF149861 |
| *Koerberiella wimmeriana* | Norway | O-L-163472 | MK812168 | — | — | — | — |
| ***Lecaimmeria botryoides*** | **China: Sichuan** | **KUN 20-66706** | **MZ227401** | **MZ227046** | **—** | **—** | **—** |
| ***L. botryoides*** | **China: Qinghai** | **KUN 20-66765** | **MZ227412** | **MZ227053** | **MZ343400** | **MZ343457** | **MZ227027** |
| ***L. botryoides*** | **China: Qinghai** | **KUN 20-66891** | **MZ227431** | **—** | **MZ343422** | **—** | **—** |
| ***L. botryoides*** | **China: Qinghai** | **KUN 20-66898** | **—** | **—** | **MZ343408** | **—** | **—** |
| ***L. botryoides*** | **China: Qinghai** | **KUN 20-66900** | **MZ227434** | **—** | **—** | **—** | **—** |
| ***L. botryoides*** | **China: Sichuan** | **KUN 20-66707** | **MZ227402** | **MZ227047** | **—** | **—** | **—** |
| ***L. botryoides*** | **China: Sichuan** | **KUN 20-66711** | **MZ227404** | **MZ227048** | **—** | **—** | **—** |
| ***L. botryoides*** | **China: Sichuan** | **KUN 20-66713** | **MZ227405** | **MZ227049** | **MZ343403** | **—** | **—** |
| ***L. botryoides*** | **China: Sichuan** | **KUN 20-66721A** | **MZ227406** | **MZ227050** | **—** | **MZ343456** | **—** |
| ***L. botryoides*** | **China: Sichuan** | **KUN 20-66730** | **MZ227410** | **MZ227051** | **—** | **—** | **—** |
| ***L. botryoides*** | **China: Sichuan** | **KUN 20-67706** | **MZ227427** | **MZ227063** | **—** | **—** | **—** |
| ***L. botryoides*** | **China: Sichuan** | **KUN 20-66683** | **MZ227484** | **MZ227045** | **—** | **—** | **—** |
| *L. iranic* | China | SDNU20117623 | KR061348 | — | — | — | — |
| *L. iranic* | China | SDNU20117663 | KR061347 | — | — | — | — |
| ***L. lygaea*** | **China: Tibet** | **KUN 20-69054** | **MZ227458** | **MZ227075** | **—** | **—** | **—** |
| ***L. lygaea*** | **China: Tibet** | **KUN 20-69070** | **MZ227459** | **MZ227076** | **—** | **—** | **—** |
| ***L. lygaea*** | **China: Tibet** | **KUN 20-69072** | **MZ227460** | **MZ227077** | **—** | **MZ343449** | **—** |
| ***L. mongolica*** | **China: Inner Mongolia** | **SDNU20190354** | **MZ227389** | **MZ227038** | **MZ343404** | **—** | **—** |
| ***L. mongolica*** | **China: Inner Mongolia** | **SDNU20190350** | **MZ227388** | **MZ227037** | **—** | **—** | **—** |
| ***L. mongolica*** | **China: Inner Mongolia** | **SDNU20117851** | **MZ227396** | **—** | **—** | **—** | **—** |
| ***L. mongolica*** | **China: Inner Mongolia** | **SDNU20117613** | **MZ227397** | **—** | **—** | **—** | **—** |
| ***L. mongolica*** | **China: Inner Mongolia** | **SDNU20117399** | **MZ227398** | **—** | **—** | **—** | **—** |
| ***L. orbicularis*** | **China: Qinghai** | **KUN 20-66886B** | **MZ227429** | **MZ227064** | **MZ343416** | **—** | **—** |
| ***L. orbicularis*** | **China: Qinghai** | **KUN 20-66896** | **MZ227432** | **MZ227065** | **MZ343402** | **—** | **—** |
| ***L. orbicularis*** | **China: Qinghai** | **KUN 20-66899** | **MZ227433** | **MZ227066** | **MZ343401** | **—** | **—** |
| ***L. orbicularis*** | **China: Qinghai** | **KUN 20-66901** | **MZ227435** | **—** | **—** | **—** | **—** |
| ***L. orbicularis*** | **China: Qinghai** | **KUN 20-66908** | **—** | **MZ227070** | **MZ343415** | **MZ343442** | **—** |
| ***L. orbicularis*** | **China: Qinghai** | **KUN 20-66909** | **—** | **—** | **MZ343394** | **MZ343446** | **—** |
| ***L. orbicularis*** | **China: Qinghai** | **KUN 20-66935** | **MZ227440** | **MZ227071** | **MZ343421** | **—** | **—** |
| ***L. orbicularis*** | **China: Qinghai** | **KUN 20-66943** | **MZ227442** | **MZ227072** | **MZ343418** | **MZ343445** | **—** |
| ***L. orbicularis*** | **China: Qinghai** | **KUN 20-66801** | **MZ227414** | **MZ227054** | **—** | **—** | **—** |
| ***L. orbicularis*** | **China: Qinghai** | **KUN 20-66803** | **MZ227415** | **MZ227055** | **—** | **—** | **—** |
| ***L. orbicularis*** | **China: Qinghai** | **KUN 20-66811** | **MZ227418** | **MZ227056** | **—** | **—** | **—** |
| ***L. orbicularis*** | **China: Qinghai** | **KUN 20-66817** | **MZ227419** | **MZ227057** | **MZ343409** | **—** | **—** |
| ***L. orbicularis*** | **China: Qinghai** | **KUN 20-66821** | **MZ227420** | **MZ227058** | **—** | **—** | **—** |
| ***L. orbicularis*** | **China: Qinghai** | **KUN 20-66826A** | **MZ227422** | **MZ227059** | **MZ343396** | **—** | **—** |
| ***L. orbicularis*** | **China: Qinghai** | **KUN 20-66829** | **MZ227423** | **MZ227060** | **MZ343395** | **—** | **—** |
| ***L. orbicularis*** | **China: Qinghai** | **KUN 20-66833** | **MZ227424** | **MZ227061** | **MZ343410** | **MZ343443** | **MZ227030** |
| ***L. orbicularis*** | **China: Qinghai** | **KUN 20-66841** | **MZ227425** | **MZ227062** | **MZ343414** | **—** | **—** |
| ***L. orbicularis*** | **China: Qinghai** | **KUN 20-66965** | **MZ227443** | **MZ227073** | **MZ343411** | **—** | **—** |
| ***L. orbicularis*** | **China: Qinghai** | **KUN 20-66979** | **MZ227445** | **MZ227074** | **MZ343407** | **MZ343448** | **—** |
| ***L. orbicularis*** | **China: Sichuan** | **KUN 20-66747** | **MZ227436** | **MZ227067** | **MZ343397** | **MZ343447** | **—** |
| ***L. orbicularis*** | **China: Sichuan** | **KUN 20-66750** | **—** | **MZ227068** | **—** | **—** | **—** |
| ***L. orbicularis*** | **China: Sichuan** | **KUN 20-66753** | **—** | **MZ227069** | **MZ343412** | **MZ343444** | **—** |
| ***L. orbicularis*** | **China: Sichuan** | **KUN 20-66743** | **MZ227411** | **MZ227052** | **—** | **—** | **—** |
| ***L. qinghaiensis*** | **China: Qinghai** | **KUN 20-3127** | **MZ227471** | **—** | **—** | **MZ343454** | **—** |
| ***L. qinghaiensis*** | **China: Qinghai** | **KUN 20-68687** | **MZ227453** | **—** | **—** | **—** | **—** |
| ***L. qinghaiensis*** | **China: Qinghai** | **KUN 20-68696** | **MZ227454** | **—** | **—** | **—** | **—** |
| ***L. qinghaiensis*** | **China: Qinghai** | **KUN 20-68698** | **MZ227455** | **—** | **MZ343420** | **MZ343450** | **—** |
| ***L. qinghaiensis*** | **China: Qinghai** | **KUN 20-3115** | **MZ227470** | **—** | **—** | **MZ343453** | **—** |
| ***L. qinghaiensis*** | **China: Qinghai** | **KUN 20-849** | **MZ227469** | **MZ227078** | **MZ343419** | **MZ343452** | **MZ227035** |
| ***L. tibetica*** | **China: Tibet** | **KUN 19-64071** | **—** | **MZ227082** | **—** | **—** | **—** |
| ***L. tibetica*** | **China: Tibet** | **KUN XY19-1288i** | **MZ227474** | **MZ227039** | **—** | **—** | **MZ227036** |
| ***L. tibetica*** | **China: Tibet** | **KUN XY19-1288A** | **MZ227475** | **MZ227040** | **—** | **—** | **—** |
| ***L. tuberculosa*** | **China: Gansu** | **KUN 18-58856** | **MZ227476** | **MZ227041** | **—** | **—** | **—** |
| ***L. tuberculosa*** | **China: Gansu** | **KUN 18-58857** | **MZ227477** | **MZ227042** | **—** | **—** | **—** |
| ***L. tuberculosa*** | **China: Gansu** | **KUN 18-58865A** | **MZ227478** | **MZ227043** | **—** | **—** | **—** |
| ***L. tuberculosa*** | **China: Gansu** | **KUN 18-59835** | **MZ227479** | **MZ227044** | **—** | **—** | **—** |
| ***L. tuberculosa*** | **China: Qinghai** | **KUN 20-68077** | **MZ227450** | **—** | **—** | **MZ343451** | **—** |
| ***L. tuberculosa*** | **China: Qinghai** | **KUN 20-68055** | **MZ227451** | **—** | **—** | **MZ343455** | **—** |
| *Lecidea atrobrunnea* | Antarctica | AAS_Convey00458 | MK620076 | — | MK684891 | HQ660524 | MK684569 |
| *L. auriculata* | Argentina | UR00061 | MK620123 | — | MK684941 | — | MK684627 |
| *L. berengeriana* | Unknown | Arup L00015 | HQ650659 | HQ660537 | — | HQ660526 | HQ660562 |
| *L. confluens* | Unknown | J. Buschbom 21.8.2001-44 | — | AY532994 | — | — | — |
| *L. confluens* | Austria | Herbarium Tuerk 39641 | — | — | — | — | GU074492 |
| *L. fuscoatra* | Sweden | Arup L02894 | HQ650662 | HQ660541 | — | HQ660528 | HQ660566 |
| *L. grisella* | Turkey | Trabzon 2 | HQ605931 | — | — | — | — |
| *L. grisella* | Sweden | Arup L02723 | — | HQ660542 | — | HQ660529 | HQ660567 |
| *L. laboriosa* | USA | F. Lutzoni et al. 03.09.05-5 | — | KJ766586 | — | DQ992432 | — |
| *L. laboriosa* | Unknown | U.C. Riverside 43266UCR1 | — | — | MK684890 | — | GU074503 |
| *L. lapicida* | USA | Lendemer 11081 | HQ650665 | HQ660544 | — | HQ660530 | HQ660570 |
| *L. lithophila* | Canada | UR00246 | MK620247 | — | MK685037 | — | MK684743 |
| *L. plana* | Sweden | LD L03170 | EU259903 | — | — | KF683111 | GU074497 |
| *L. promiscens* | Argentina | UR00129 | KX120205 | — | MK684964 | — | MK684661 |
| *L. silacea* | Unknown | AFTOL-ID 1368 | HQ650629 | — | DQ986820 | DQ992431 | — |
| *L. silacea* | Unknown | Wedin 6865 | — | AY756340 | — | — | — |
| *L. silacea* | Unknown | Herbarium Tuerk 42156 | — | — | — | — | GU074496 |
| *L. tessellata* | Unknown | Lay 01-0360 | HQ650671 | HQ660548 | — | — | — |
| *L. uniformis* | USA | Hollinger 6775 | NR_158514 | KY800515 | — | — | — |
| *Lecidoma demissum* | Unknown | AFTOL 1376 | HQ650630 | — | KJ766867 | DQ992445 | — |
| *Pachyphysis ozarkana* | Unknown | J. Buschbom 11.10.1997-2 | — | AY532988 | — | — | — |
| *Poeltidea cf. perusta* | Chile | UR00026 | MK620106 | — | MK684886 | — | MK684599 |
| *P. perusta* | Chile | UR00039 | MK620112 | — | MK684888 | — | MK684609 |
| *Porpidia albocaerulescens* | USA | Tripp 2279 | KJ653475 | — | — | DQ992443 | — |
| *P. cinereoatra* | United Kingdom | Orange 20432 | KJ162305 | — | — | — | — |
| *P. cinereoatra* | Unknown | J. Buschbom 30.8.2001-1 | — | AY532941 | — | — | — |
| *P. cinereoatra* | Unknown | J. Guccion 1187 | — | — | — | — | KF683104 |
| *P. contraponenda* | United Kingdom | Orange 20447 | KJ162297 | — | — | — | — |
| *P. contraponenda* | Unknown | K. Glew 000810-2 | — | AY532942 | — | — | — |
| *P. degelii* | USA | Tripp 2503 | KJ653479 | — | — | — | KJ653473 |
| *P. grisea* | China | SDNU YN0116 | KY509524 | — | — | — | — |
| *P. grisea* | Unknown | J. Buschbom 25.8.1999-15 | — | AY532951 | — | — | — |
| *P. hydrophila* | United Kingdom | Orange 16218 | KJ162317 | — | — | — | — |
| *P. hyposticta* | China | SDNU:20141385 | NR_156592 | — | — | — | — |
| *P. macrocarpa* | Austria | Herbarium Tuerk 39740 | EU263923 | — | — | — | GU074512 |
| *P. macrocarpa* | Austria | UR00411 | MK620258 | — | MK685056 | — | MK684730 |
| *P. speirea* | China | SDNU20140742 | KY509523 | — | — | — | — |
| *Rhizocarpon disporum* | Unknown | Lutzoni 96.10.26-9 st.1 (1/2) | — | AF356678 | — | AY641069 | — |
| *R. geographicum* | Norway | Ihlen 941 | AF483619 | AY853389 | — | — | AF483187 |
| *Romjularia lurida* | Spain | S. Pérez-Ortega 1372 | KF683091 | KF683107 | EF524328 | — | — |
